# Comparing the development of cortex-wide gene expression patterns between two species in a common reference frame

**DOI:** 10.1073/pnas.2113896119

**Published:** 2022-10-06

**Authors:** Sebastian S. James, Mackenzie Englund, Riley Bottom, Roberto Perez, Kathleen E. Conner, Kelly J. Huffman, Stuart P. Wilson, Leah A. Krubitzer

**Affiliations:** ^a^Department of Psychology, University of Sheffield, Sheffield S10 2TN, United Kingdom;; ^b^Department of Psychology, University of California Davis, Davis, CA 95616;; ^c^Department of Psychology, University of California Riverside, Riverside, CA 92521

**Keywords:** neocortex, brain evolution, brain development, evo-devo, cortical arealization

## Abstract

Advances in sequencing techniques have made comparative studies of gene expression a current focus for understanding evolutionary and developmental processes. However, insights into the spatial expression of genes have been limited by a lack of robust methodology. To overcome this obstacle, we developed methods and software tools for quantifying and comparing tissue-wide spatial patterns of gene expression within and between species. Here, we compare cortex-wide expression of *RZRβ* and *Id2* mRNA across early postnatal development in mice and voles. We show that patterns of *RZRβ* expression in neocortical layer 4 are highly conserved between species but develop rapidly in voles and much more gradually in mice, who show a marked expansion in the relative size of the putative primary visual area across the first postnatal week. Patterns of *Id2* expression, by contrast, emerge in a dynamic and layer-specific sequence that is consistent between the two species. We suggest that these differences in the development of neocortical patterning reflect the independent evolution of brains, bodies, and sensory systems in the 35 million years since their last common ancestor.

## Significance

We created new software tools for comparing spatial patterns of gene expression between the brains of different species in three dimensions. Using these tools, we show how cortex-wide patterns of mRNA expression are conserved between developing species, focusing on two genes involved in defining cortical fields. Specifically, *Id2* expression patterns developed in a layer-by-layer sequence that was highly conserved between mice and voles, whereas *RZRβ* expression developed more gradually in mice, particularly in the primary visual area, which doubled in size over the first postnatal week. These findings demonstrate the usefulness of our open-source software for allowing scientists from many fields to quantify where and when genes are expressed across development and between species.

Almost everything we know about the human brain comes from comparative studies of other animals: from genes involved in cortical development to system-level networks that generate complex behaviors. Comparative studies of living species provide a robust means by which to understand unknown forms, like humans, and even extinct forms like our early mammalian ancestors. Importantly, these types of studies are critical for identifying features of brain organization that are conserved between species and those that may have been derived in different lineages. They also allow us to determine how developmental programs and timing schedules may vary across species, and to better understand how phenotypic diversity can be generated over shorter and longer timescales. Finally, by making valid comparisons across species, we can begin to understand how complexity emerges in different nervous systems, the rules of brain construction, and the constraints imposed on developing and evolving nervous systems.

Despite the importance of comparative studies in biology, most comparisons of anatomically reconstructed data are subjective, and most gene sequencing studies neglect the actual spatial patterns of gene expression across a structure, focusing instead on cell-type expression (1–3). Moreover, many current methods for making comparisons fail to capture the three-dimensional nature of the brain, which is composed of asymmetrical structures that can vary markedly in relative shape, size, and location across species and between developmental time-points. Despite the three-dimensional (3D) nature of the brain, most studies collapse data into two dimensions driven largely by the plane of section at which the brain is cut.

As such, neurobiologists are faced with two challenges. First, attempting to understand 3D structures by analyzing two-dimensional (2D) images is inherently problematic because the loss of spatial information is unavoidable, especially in curved structures (4). 2D analysis often involves prespecifying regions of interest (ROIs) to quantify the presence of labeled cells or mRNA expression after in-situ hybridization (ISH), narrowing the focus and potentially missing overall differences across a structure, such as the neocortex. A second challenge, which arises when making comparisons between structures in different species and/or at different developmental time-points, or between different experimental conditions, is determining the extent to which 2D spatial patterning might be invariant to basic transformations in the size and shape of the 3D structure. To this end, it is important for comparisons to be made with respect to a common anatomical reference frame.

In the current study, we overcame these challenges by developing a set of algorithms for brain slice registration in 3D, and for incorporating ISH data into a common reference frame to enable point-by-point comparisons between species or experimental conditions (see *Materials and Methods*). These tools, which we refer to collectively as *Stalefish, the Spatial Analysis of Fluorescent (and nonfluorescent) In-Situ Hybridization*, allowed for the laminar and spatial patterns of expression of genes involved in cortical development to be quantified and compared in two species of age matched rodents across early postnatal development. Our analysis of *Id2* and *RZRβ* cortical expression patterns in mouse and vole brains reveals both a strong layer-specific conservation of the patterning of these genes, as well as area-specific differences in development that shed new light on the ontogeny and phylogeny of neocortical arealization.

## Reconstructing Whole-Brain Patterns of Gene Expression From Processed Tissue

**Fig. 1.**
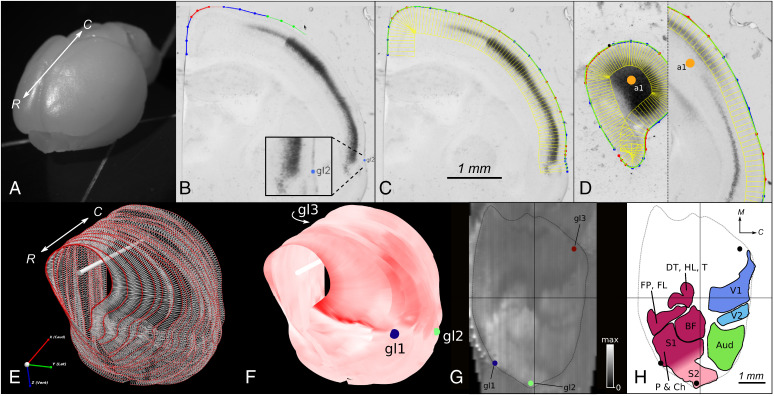
*Stalefish* workflow. (*A*) Rostral view of an unfixed vole brain. The rostral-caudal axis is shown (white arrow). (*B*–*D*) Screenshots from *Stalefish* of coronal sections of *RZRβ*-hybridized vole tissue showing the curve drawing method. Dark regions indicate a high expression of *RZRβ* mRNA. (*B*) Marked locations around the perimeter of the brain. The perimeter points are collected into small sets of 4 or 5 points at a time. The green points are the newest set of perimeter locations and will become the next ‘red’ set (the red and blue colors are simply a guide for the user). The number of points in each section determines the order of the Bezier curve which will be fitted to that section. The blue dot labelled ‘gl2’ shows a global landmark placement for transformations. (*C*) Once the perimeter points have been laid out, a piecewise fit is found for the points by modifying the individual Bezier curves to ensure that the curve gradient is continuous at the joints. The green line shows the final fit. Evenly spaced normal vectors extended down from the fit line give sampling boxes in yellow. (*D*) An axis mark (orange dot) on the first and last slice define a brain axis for digital alignment. (*E*) *Stalefish* output using sfview. Curve points are shown as red spheres. By connecting the spheres to make a mesh, a surface is generated. The white bar shows the user-defined brain axis. The rostral-caudal axis is shown (white arrow). (*F*) The mean luminance of the sampling boxes can then be displayed on the smoothed surface to give a 3D reconstruction of the gene expression. Here, we used a monochrome colormap for which full-saturation red corresponds to the maximum *RZRβ* expression signal. (*G*) Digitally flattened and reference-frame transformed 3D surface map (from *B*–*F*) using sfview. (*H*) Freehand loops drawn around the identifiable regions of expression in (*G*). Areas (mm^2^) are: V1 1.78; V2 0.49; Aud 1.74; FP/FL 0.71; DT/HL 0.58; BF 1.16; P/C S1 and S2: 3.07. Neocortical area (dotted line): 23.9. Abbreviations: rostral (R), caudal (C), medial (M), barrel field (BF), dorsal trunk (DT), hind leg (HL), tail (T), forepaw (FP), forelimb (FL), perioral (P), chin (Ch).

**Fig. 2.**
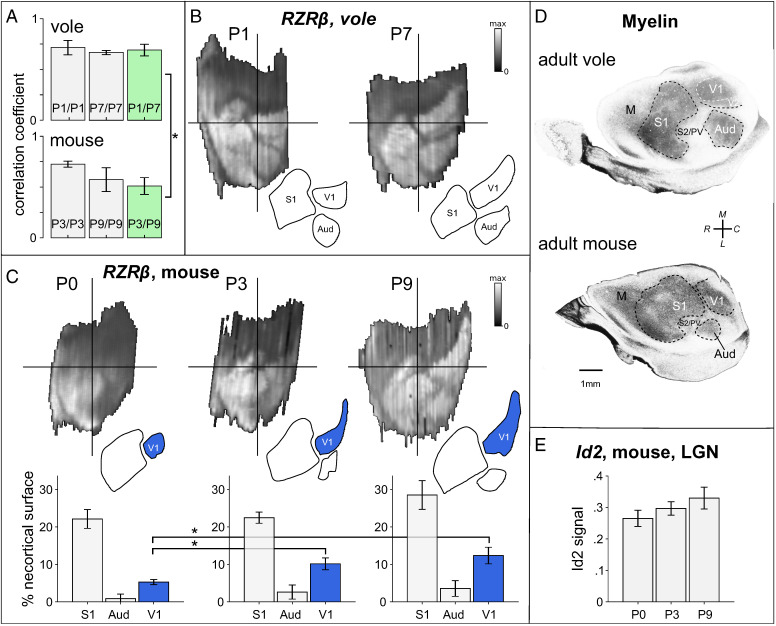
Highly correlated patterns of *RZRβ* expression between species develop at different rates. (*A*) Bar graphs showing the correlation between (and among) digitally reconstructed *RZRβ* expression maps obtained from layer 4 of young (P1) and older (P7) voles (*Top*), and of young (P3) and older (P9) mice age-matched by postconception day. Maps are strongly correlated between species and over time, but are significantly less correlated over time in mice (green bars). (*B*) Example layer 4 *RZRβ* expression maps digitally reconstructed from a P1 and a P7 vole. At both ages, *RZRβ* expression levels are high in spatially distinct regions whose shapes and locations correspond to those of adult neocortical fields (including the S1, V1, Aud, V2, S2, and PV). (*C*) Example layer 4 *RZRβ* expression maps obtained from a P0, P3, and a P9 mouse. The patterns of expression come into focus across developmental time, with an area of high expression at the location corresponding to putative V1 clearly increasing in expression strength and growing in size over time. Below each example is a bar graph showing the area of the region of high expression corresponding to the primary sensory fields. Compared to P0, the putative V1 (blue bars) was significantly larger at P3 and at P9, relative to the size of the neocortex. (*D*) Flattened cortical sections stained for myelin in an adult vole (*Top*) and mouse (*Bottom*) showing cortical field boundaries. (*E*) A nonsignificant trend in the levels of expression of *Id2* in digitally reconstructed LGN was for a small increase in the mean expression level over time.

In mice (*Mus musculus)* and prairie voles (*Microtus ochrogaster*), direct layer- and area-specific comparisons were made between the cortex-wide expression patterns of two genes important for cortical development: *RZRβ* (RAR-related orphan receptor beta), and *Id2* (inhibitor of DNA-binding 2) (5–9). Using an algorithm for slice registration developed as part of the *Stalefish* methodology, we were able to visualize and quantify cortex-wide layer-specific patterns of gene expression in 3D and determine the extent to which the development of these patterns is conserved across species (Fig. 1). We concentrated our efforts on relating patterns of gene expression to the primary somatosensory area (S1), the primary visual area (V1) and auditory cortex (Aud), which contains several fields including the primary auditory area and the anterior auditory field [see ref. (10) for a review]. In adults, these functional areas correspond to architectonically distinct, darkly myelinated subdivisions (Fig. 2).

To illustrate how layer-specific cortical in-situ hybridization (ISH) data can be reconstructed from coronal sections, the *Stalefish* curve drawing tool was used to process an entire hemisphere of a postnatal day one (P1) vole brain (Fig. 1*A*) hybridized for *RZRβ* (Fig. 1 *B*–*D*). The *Stalefish* 3D viewer tool was then used to show the 3D-reconstructed expression pattern in layer 4 (Fig. 1 *E* and *F*). Finally, the *Stalefish* digital flattening tool was used to project the data into a new 2D plane for subsequent analysis (Fig. 1*G*). The shapes and positions of several areas of high and low expression in the neonatal vole cortex were revealed to correspond well with descriptions of cortical field boundaries later observed in adult voles (11) (Figs. 1*H* and 2), confirming the proposed role of this gene as an area marker for putative neocortical fields. The *Stalefish* methodology was further validated by comparing reconstructions of our mouse data to reconstructions that we made using similar data obtained from the Allen Mouse Brain Atlas (12, 13) (*SI Appendix*, Fig. S1).

## Highly Correlated Patterns of *RZR****β*** Expression between Species Develop at Different Rates

We sought to quantify the extent to which spatial patterns of expression in two neocortical area marker genes (*RZRβ* and *Id2*) vary between species and during early postnatal development. For each marker, we hybridized brain tissue from mice at postnatal days P0 (*n* = 3 per marker), P3 (*n* = 3 per marker), and P9 (*n* = 4 per marker), and from Prairie voles at postnatal days P1 (*n* = 7 per marker) and P7 (*n* = 3 per marker). Species were age matched in terms of both postconception days and early developmental cortical events (such as the emergence of barrels) (14). P1 voles and P3 mice are age-matched (both 23 d postconception), as are P7 voles and P9 mice (both 29 d postconception) (15). Because tissue would have to be collected in utero we did not collect vole tissue that is age matched to P0 mouse. However, we included data from the P0 mouse to investigate the temporal trajectory of expression patterns across three important time-points in this species.

First we examined the patterns of expression of *RZRβ*, a purported primary sensory area marker whose high expression in layer 4 is heavily influenced by input from thalamic sensory nuclei (9, 16). Because *RZRβ* is highly expressed in this layer, and sparse in other layers at early stages of development, we restricted our analysis of *RZRβ* to layer 4 (Fig. 2) (17).

We compared reconstructed *RZRβ* cortical expression maps to flattened cortical sections of adult animals stained for myelin, which show clear delineations of cortical field boundaries, and found that *RZRβ* expression in the early postnatal brains revealed the putative cortical areas later delineated by myelin (10, 18). Specifically, reconstructed *RZRβ* expression patterns clearly demarcated the cortical field boundaries of putative S1, V1, and Aud in both P1 and P7 voles (Fig. 2*B*), and most clearly demarcated these areas in mice from P3 onwards (Fig. 2*C*). From the earliest time tested in both species, the subregions representing the map of the entire body could be identified from the expression patterns in putative S1 (19, 20). The second somatosensory/parietal ventral area (S2/PV) was also delineated in both species. Furthermore, in voles, *RZRβ* expression patterns already displayed a distinct boundary between the primary and second visual areas (V1 and V2) at P1, whereas this delineation did not become distinct in mice until between P3 and P9. To our knowledge, evidence that distinct gene expression patterns delineate higher-order cortical areas has not been demonstrated at such an early stage in development: This suggests that early area-specific genes involved in the establishment of thalamocortical innervation may play a stronger role in patterning higher-order areas than has been previously thought (21).

To quantify the differences between species across development, we used the positions of external anatomical landmarks commonly identifiable in all brains, and then computed a linear transformation by mapping the flattened reconstruction of each brain to the coordinate system of an arbitrary reference brain in a given comparison set. This allowed cortex-wide expression patterns from multiple brains to be compared point-by-point in a common reference frame (see *Materials and Methods*). Following these transformations, we quantified the similarity of cortex-wide patterns of *RZRβ* expression by applying Pearson correlation analyses to the point-by-point matched expression levels across pairs of maps, excluding any points not present in every pattern submitted for a given set of comparisons.

First, we correlated the layer 4 *RZRβ* maps obtained from P3 mice with those obtained from P1 voles, and correlated the layer 4 *RZRβ* maps from P9 mice with those obtained from P7 voles. *RZRβ* expression maps were strongly correlated between species at the earlier (r_avg_ = 0.659 ± 0.090) and later (r_avg_ = 0.457 ± 0.044) time-points (Fig. 2 *A*, green bars). However, these between-species correlations were significantly reduced over time (*t* (32) = 7.045, *P* < 0.001). This reduction in correlation between earlier and later time-points suggests that species-specific *RZRβ* expression patterns begin to emerge over the first postnatal week. Next, in mice we correlated the layer 4 *RZRβ* maps obtained at P3 with those obtained at P9, and in voles we correlated layer 4 *RZRβ* maps obtained at P1 with those obtained at P7. While the maps were strongly correlated across time for mice (r_avg_ = 0.509 ± 0.083) and voles (r_avg_ = 0.691 ± 0.057), the conservation of the map patterns between time-points was significantly reduced in mice compared to voles (*t* (32) = 7.195, *P* < 0.001). Together these analyses suggest that while patterns of *RZRβ* expression are overall highly consistent between species, the patterning of mouse maps becomes more distinct over early postnatal development while patterning of vole maps is consistently distinct across postnatal development. Thus, cortex-wide *RZRβ* expression is dynamically regulated across early postnatal development in mice but in voles appears to be stabilized by P1.

Visual inspection of the individual maps in mice of different ages suggested that the time-dependent variability in mouse maps may be largely due to a developmental increase in the size of the region that corresponds to putative V1. To investigate this possibility, we used the *Stalefish* freehand drawing tool to trace the boundaries of high expression in the locations corresponding to putative S1, Aud, and V1, and to estimate the size (as percentages of the entire neocortical surface area) of each delineated region (Fig. 2*C*). A multivariate ANOVA revealed a significant effect of developmental time (P0, P3, P9) on the relative size of the region corresponding to putative V1 (*F*(2,7) = 10.830, *P* = 0.007), and no such effects in putative S1 or Aud. This effect is attributable to differences in the proportion of the neocortical surface over which *RZRβ* expression is high in putative V1 between P0 (5.273 ± 0.673%) and P3 (10.121 ± 1.566%) mice (*t*(4) = −4.022, *P* = 0.016), and between P0 and P9 (12.328 ± 2.197%) mice (*t*(5) = −4.543, *P* = 0.006). This region was found to double in relative size over the first 3 postnatal days and then to continue expanding more slowly over the subsequent postnatal week. While the effect of postnatal age was not significant in Aud or S1, the trend we observed in each region was for a relative increase in their size over time (Fig. 2*C*). To determine if these temporal differences in expression of *RZRβ* across development that we observed in visual cortex of the mouse were also present in the visual thalamus we examined the expression level of *Id2* and *RZRβ* in the lateral geniculate nucleus (LGN) at the same three developmental time-points. Note that by comparison to the observations in putative V1 of the mouse, the expression level of *Id2* in LGN between P0, P3, and P9, although trending toward an increase with age, did not differ significantly, and nor did the expression of *RZRβ* (Fig. 2). This lends support to the idea of mosaic evolution, as opposed to concerted evolution, at least for these genes, i.e., with alterations to the cortex seeming to arise independently of the rest of the brain (22).

Overall, our analyses of patterns of *RZRβ* expression in the developing neocortex delineate the putative primary fields in a way that is highly conserved between species over the long time course of evolution. However, expression patterns in mice develop differently compared to voles, with a slower emergence of distinct patterns associated with primary cortical fields over a prolonged postnatal period.

## Layer-Specific Development of Id2 in the Neocortex

Next, between species and across developmental time, we compared neocortical patterns of expression of *Id2*, a key transcription factor in neurodevelopment that regulates cellular differentiation and neurite outgrowth (23–26). *Id2* was found to be expressed in all cortical layers except layer 4 in both species, similar to findings from previous studies (27). *Stalefish* was therefore used to fit curves on each *Id2*-hybridized section of the series at depths from the neocortical surface that correspond to layer 2/3, layer 5, and layer 6 (henceforth L2/3, L5, and L6), and to digitally unwrap and flatten the 3D reconstructed expression patterns. While the patterns appeared highly consistent between voles and mice at a given time-point, and within a given cortical layer, we observed marked differences in the patterns of expression between time-points and between layers in both species.

To quantify these observations, we used the *Id2* expression maps in L2/3 as a reference, and obtained point-by-point correlation coefficients for each pair of L5 and L2/3 maps, and for each pair of L6 and L2/3 maps. Then we compared those for L5 with those for L6 between the two species and between the two developmental time-points (P0 mouse data were not included in this analysis). A three-way ANOVA, with species (mouse vs. vole), developmental time (young, P3/P1, vs. older, P9/P7), and layer (L5 vs. L6) as factors, revealed significant effects of development (*F*(1,144) = 66.80, *P* < 0.001) and layer (*F*(1,144) = 45.87, *P* = 0.003) on the correlations with the L2/3 reference maps. L5 and L6 maps were more strongly correlated with L2/3 maps in older animals (r_avg_ [P7 vole L5] = 0.373 ± 0.202; r_avg_ [P7 vole L6] = 0.047 ± 0.232; r_avg_ [P9 mouse L5] = 0.386 ± 0.217; r_avg_ [P9 mouse L6] = 0.156 ± 0.269) than in younger animals (r_avg_ [P1 vole L5] = 0.141 ± 0.217; r_avg_ [P1 vole L6] = −0.293 ± 0.224; r_avg_ [P3 mouse L5] = −0.064 ± 0.129; r_avg_ [P3 mouse L6] = −0.297 ± 0.128), and these correlations were stronger for L5 maps than for L6 maps. No significant differences between species, or interactions among species, time, and layer were found. Independent *t* tests revealed significant differences in correlations with the L2/3 reference maps across development, in mouse L5 (*t* (16) = −5.35, *P* < 0.001) and mouse L6 (*t* (16) = −4.57, *P* < 0.001), and in vole L5 (*t*(56) = −2.99, *P* = 0.004) and vole L6 (*t*(56) = −4.167, *P* < 0.001), as well as significant differences between the two layers in mice at P3 (*t* (16) = 3.84, *P* = 0.001) and in voles at P1 (*t*(96) = 9.75, *P* < 0.001) and P7 (*t*(96) = 3.18, *P* = 0.006).

**Fig. 3.**
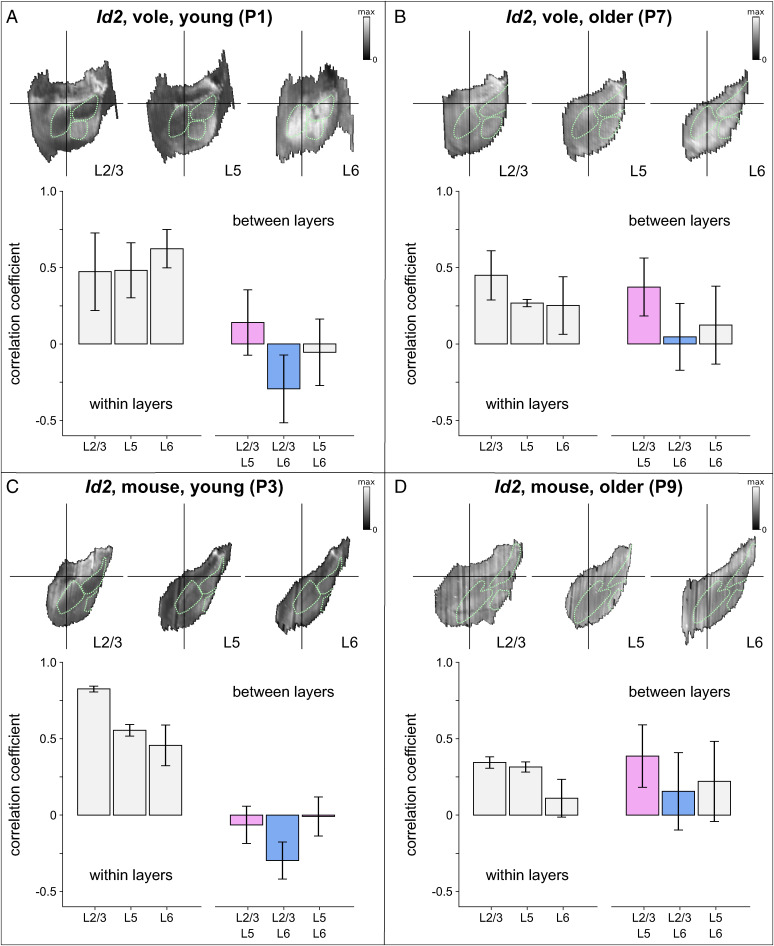
Layer-specific development of expression patterns of *Id2* in mice and voles. Digitally reconstructed patterns of *Id2* expression were obtained from (*A*) P1 voles, (*B*) P7 voles, (*C*) P3 mice, and (*D*) P9 mice, at depths that corresponded to layer 2/3, layer 5, and layer 6. In each panel the *Top Row* shows an example of an *Id2* expression map for each cortical layer, and the bar graphs show correlations between all maps measured from within a given layer (*Left*) and for each pair of maps measured from two different layers (*Right*). Bars colored pink (showing correlations between layer 5 maps and layer 2/3 maps) and blue (showing correlations between layer 6 and layer 2/3 maps) represent data that were submitted for the main analysis. This analysis revealed distinct layer-specific changes in map development that was highly consistent between the two species. Layer 5 and layer 2/3 map patterns are uncorrelated in early postnatal development and become correlated over time, whereas layer 6 and layer 2/3 map patterns are anti-correlated in early postnatal development and become uncorrelated over time. Green dotted lines show outlines of the presumptive S1, V1, Aud, obtained by digitally tracing regions of high *RZRβ* expression from one example brain in each of the four age/species combinations.

Overall, these analyses show that L5 and L2/3 *Id2* expression maps are uncorrelated in younger mice and voles and become correlated in both species with age, and that L6 and L2/3 *Id2* expression maps are anti-correlated in younger animals and become uncorrelated during development. While the patterns of *Id2* expression in L6 are more variable among older mice than older voles, these layer-specific developmental changes occur consistently between the two species, as supported by the lack of an overall interaction effect. The anti-correlation between L6 and L2/3 maps early in development in both species is the result of a distinct lack of expression in L2/3 but high L6 expression in the putative primary cortical areas. However, at the later time-point, L2/3 expression increases in primary fields, and thus becomes more correlated with L6 expression (Fig. 3).

These findings constitute a quantitatively robust demonstration of evolutionarily conserved layer-specific developmental changes in the expression patterns of what appears to be a distinct neocortical area marker. Together with the findings from *RZRβ*, our data describe the development of neocortical area-marker expression patterns as a highly dynamic, yet highly conserved, layer-specific process with a rapid onset and stabilization in voles, and a more prolonged trajectory in mice in layer 4, which otherwise unfolds in a similar sequence. The relative speed of the development of *RZRβ* patterning in layer 4 may be one of several important factors in establishing species differences in neocortical arealization, such as the prominence of the secondary visual area seen in neonatal voles.

## Conclusions

Our results indicate that cortex-wide gene expression unfolds in early postnatal life in mice and voles in a spatially similar, but temporally different manner. In both species, high expression patterns of *RZRβ* and *Id2* were clearly related to the putative primary cortical areas based on their location, and overall size and shape as they related to the primary cortical areas defined in adults. Altogether, cortex-wide correlations of gene expression between species show the highly conserved spatial and laminar patterns of expression in mice and voles, and also show how these spatially similar patterns emerged and stabilized at different rates in each species.

While it is possible that these differences are due to the fact that laboratory mice are highly inbred, and thus may have derived patterns of expression compared to other rodents, we believe the observed differences reflect the independent evolution of brains, bodies, and life histories of voles and mice, whose ancestors diverged some 35 million years ago (28). For example, voles are crepuscular with seasonal changes in activity periods while mice are nocturnal; these differences may have a large impact on visual cortex organization, and function, particularly on temporal aspects of development. On the other hand, both mice and voles have an elaborate barrel cortex and actively engage in whisking behaviors as a central component of exploration (19, 29, 30). However, their respective social structures and early life parental care differ markedly. Prairie voles are monogamous and biparental, live in extended family groups and have a complex acoustic communication system (31, 32). Mice, on the other hand, are promiscuous, alloparental, and colonial, with a more limited repertoire of vocal communication. While differences in early life experience have been shown to alter cortical organization and gene expression in both mice and voles (17), it is unlikely that the differences in cortical organization that we observed are purely experience-driven because our data are from neonatal animals with limited exposure to external environmental stimuli. Instead, we posit that the observed variation is due to evolutionary changes to sensory arrays, body morphology, and the neurodevelopmental program, which serves as a scaffold for the respective social and physical environment that voles and mice experience in early postnatal life and beyond (33). These differences in lifestyle and use of sensory systems likely coevolved with alterations in sensory areas of the neocortex, orchestrated by transcription factors such as *Id2* and *RZRβ,* which are associated with the formation and transcriptional identity of cortical layers and areas during neurodevelopment (9, 24–26, 34).

The *Stalefish* software tools were designed to facilitate the comparison of surface expression patterns between species and across development, from 3D reconstruction to point-by-point comparisons between tissues. While the specific application of those tools presented here is particularly suited to elucidating species differences in neocortical development, the component tools for sampling images along user-defined curves and for interactively visualizing 3D surfaces etc., were designed to be highly flexible. They can, for example, easily be applied to study laminar expression profiles at arbitrary resolution through the cortical depth. The strong correlations in *Id2* expression between L2/3 and L5 in older animals shown in Fig. 2, and strong anti-correlations between L2/3 and L6 for younger animals, confirm high laminar specificity for this gene, especially given the absence of signal in the L4 region that separates them. However, our methods for data collection and data storage also allow laminar expression profiles to be reconstructed at much higher spatial resolution through the cortical depth (*SI Appendix*, Figs. S2–S4). These tools can also be applied to study other brain structures whose spatial patterns of gene expression have eluded quantitative study for decades, such as the hippocampus and dorsal thalamus (*SI Appendix*, Figs. S5 and S6). Moreover, the *Stalefish* tools can readily be applied in different combinations, interfaced with third-party software, and extended to incorporate new features.

A potentially useful extension is to allow anatomical landmarks from the reference atlas of a model species to be imported into a *Stalefish* project to guide data collection. This extension was straightforward to develop and when we applied this technique to sample our data from regions in an Allen mouse brain atlas identified as barrel cortex and V1, we found a general pattern of elevated expression for *Id2* and *RZRβ* in both species (*SI Appendix*, Fig. S7). We also developed an extension of the core application to allow nonuniform sampling bins to be specified along the length of the curve traced for each slice, to account for possible effects of variations in the tangential profile of a reconstructed surface, though this did not improve the quality of reconstructed patterns, even for the intricately folded hippocampal formation (*SI Appendix*, Fig. S8). *SI Appendix*, Fig. S9 shows how these two extensions can be combined to reconstruct serial two-photon tomography imaging signal in mouse layer 4 at the resolution of individual barrel columns, demonstrating the precision and laminar specificity of reconstructions that can be achieved with these techniques. Interestingly, while we noticed stippling in the putative barrel field in our P9 mouse data when sampled at similar resolution, the expression pattern of *RZRβ* appeared not to delineate individual barrels. Further extensions to incorporate image-processing techniques, as well as techniques for automating the process of ROI identification, may yet clarify the picture.

Reference atlases are available for standard species like mouse, and can be readily ported into *Stalefish* to guide the user during data collection. Further refinements to the techniques we developed, to register newly collected gene expression data like ours to the rich anatomical data collected through existing community projects with higher fidelity, may reveal yet further insights into neocortical development in such species. However, it will in many cases be inappropriate to analyze the brain structures of one species in any absolute coordinate system defined for another. By linearly transforming one brain into the reference plane of another, and then correlating the two point-by-point, we were effectively able to pose the question: “How similar are expression patterns between two brains after accounting for differences in brain size?” For mice and voles the corresponding analysis revealed remarkably strong similarities overall, even across species and across development, suggesting that the overall expression patterns of these area-markers do scale linearly with brain size. The species differences present in our data, particularly the developmental expansion of *RZRβ* expression in putative mouse V1, were found to be localized to specific areas of the tissue, and thus are unlikely to have originated from any nonlinear deformations of the overall shapes of the cortical surfaces. It is possible that our correlation measures could be sensitive to local distortions of the tissue in the event that such distortions are in turn correlated with areas of high or low gene expression.

In sum, our study highlights the usefulness of *Stalefish* for analyzing whole brain regions, for observing overall patterns, and for allowing the scientist to then focus on ROIs (*SI Appendix*, Fig. S10) in search of the factors that drive strong and weak correlations, i.e., the factors underlying species similarities and differences. Specifically, the *Stalefish* tools enabled us to clearly observe that *RZRβ* is expressed in higher-order cortical areas such as S2/PV and V2, a discovery that would not have been possible with traditional analyses.

It would be of great interest to catalog the emergence of these higher order areas along with how patterns of expression of other genes involved in cortical development change across the first postnatal weeks, to study species or experimental differences in developmental trajectories in quantitative terms and at the level of entire neural structures. The *Stalefish* algorithms and software tools are readily applicable for the analyses of multiple species over multiple postnatal days, to elucidate where and when developmental processes are conserved or have diverged in evolution. Further, they allow for the study of how variation emerges across development and across species. While we present our own data using this new methodology here, we believe that neuroscientists and biologists will be able to utilize *Stalefish* to quantify spatial patterns of gene expression (or histological markers) in a variety of brains of different shapes, sizes and levels of complexity, and be able to build on this approach to address longstanding evolutionary and developmental questions, generating novel comparisons and deriving unique insights that may otherwise remain elusive.

## Materials and Methods

### Subjects.

Twenty prairie voles (*Microtus ochrogaster*) and 19 C57/BL6 Mice (*Mus musculus*) were used for ISH experiments. Voles were obtained through the breeding colony at the University of California Davis, and mice were obtained through the breeding colony at the University of California Riverside. All experimental procedures were approved by University of California Davis IACUC and UC Riverside IACUC and conform to NIH guidelines.

### Brain Collection.

Animals were euthanized by an overdose of sodium pentobarbital (>100 mg/kg, 390 mg/mL) and perfused with 0.1 M phosphate buffered saline (PBS) followed by 4% paraformaldehyde (PFA) in 0.1 M phosphate buffer. Brains were extracted under microscope guidance and stored in 4% PFA before being shipped to University of California Riverside for processing. Brains were then dehydrated in ascending concentrations of methanol and stored in 100% methanol at −20 °C. Brains were fixed in gelatin-albumin solution and sliced on a vibratome at 100 μm. Alignment landmarks for use in postprocessing were created by positioning a straight 21-gauge needle through the mold in which brains were fixed in the gelatin-albumin solution (dissolved in 1× PBS and fixed with 25% glutaraldehyde). Needles were removed once the gelatin-albumin fixing medium had solidified, leaving circular holes in each slice that aid the subsequent alignment process (*SI Appendix*, *SI Methods* and Fig. S1).

### ISH.

Previously established protocols for nonradioactive free-floating RNA ISH were used to assess patterns of gene expression in mice and voles (19, 29). Probes for *RZRβ* and *Id2* were applied to alternating and/or serial sections of 100 μm coronal slices. After hybridization, sections were permeabilized in 50% glycerol, mounted onto glass slides, and cover-slipped. All hybridized sections were digitally imaged using a Zeiss SteREO Discovery V.12 dissecting microscope and captured using a digital high-resolution Zeiss Axio camera (HRm) using Axiovision software (version 4.7). To account for differences in the overall illumination of slices that were imaged at different orientations to the camera, we made copies of each image and blurred them using a very wide Gaussian kernel before subtracting the blurred images from the originals to leave the signal (*SI Appendix*, *SI Methods*).

### Overview of 3D Reconstruction, Digital Unwrapping, and Point-by-Point Alignment.

In general terms, the 3D reconstruction process consisted of 1) fitting curves to the elements of a common anatomical surface that are visible across multiple 2D slice images; 2) sampling image luminance values in contiguous rectangular bins oriented tangential to each curve at evenly spaced points along its length; and 3) aligning the data obtained with respect to each curve to form a 3D surface that corresponds to the shape of the original anatomical surface from which the curves were derived. The presliced alignment within the stack of slice images was then approximated using an algorithm that aligns each curve with respect to the curve obtained from the adjacent slice, utilizing, if necessary, any available alignment marks, e.g., circular holes left by a needle. Each 3D surface was then digitally unwrapped with respect to a user defined brain axis and an angle about this axis that formed a center line through the surface (Fig. 1 and *SI Appendix*, *SI Methods*), allowing the curves to be digitally straightened while clamped to the center line. Finally, each resulting 2D expression map was linearly transformed so that its coordinates matched those of a comparable reference map obtained from another animal. This was achieved by marking three external anatomically defined locations for each structure near to the curve surface elements on selected brain slices and then, following reconstruction and digital flattening, finding the 2D linear transform to project this triplet of coordinates onto the corresponding triplet on the reference map. The linear transform was then applied to all of the sampling bin locations in the dataset for each individual, resulting in a map for each that was composed of irregularly sized, quadrilateral data pixels. These data were then resampled, using elliptical Gaussian kernels, onto a Cartesian grid of square pixels, to form a 2D matrix of binned luminance values that could be compared to similarly resampled data from the reference map on a point-by-point basis.

### Curve Fitting and Image Sampling.

To allow the researcher to define arbitrarily shaped, smooth curves that follow anatomical feature lines such as those shown in Fig. 1 *B*–*D*, we utilized the Bezier curve, a form of polynomial curve commonly used in drawing software, which is typically defined by a start and end location and a series of user-editable control points that define its curvature. While these control points (which typically lie away from the curve) give great flexibility for drawing applications, we designed the *Stalefish* curve drawing tool to exploit the fact that it is also possible to analytically determine a Bezier curve that best fits a given sequence of points. This allowed us to mark points along the boundary of an anatomical structure in each slice without considering where the Bezier control points should lie. The number of user points in the sequence determined the order of the polynomial which formed the Bezier curve, with three points specifying a quartic curve, four specifying a cubic, and n+1 points in general specifying an n-th order curve. Fits of the highest quality were obtained for our data by using multiple, low-order curves, joined end-to-end using a simple routine to modify the control points closest to the join to match the gradient at the end of one to the gradient at the start of the other. Once the (multisegmented) curves were defined by the user, N sample boxes were automatically defined by drawing N+1 equally spaced vectors along the curve and normal to it. To find N+1 equally spaced locations on the curve, the following three-step procedure was carried out numerically: First, the distance between the first and last points on the curve was computed and divided by N to get a candidate spacing, *s*. Second, up to N times, a Euclidean distance was advanced *s* along the curve, recording the coordinate at each step. Noting that Bezier curves are parameterized with *t* in the range [0,1] (mapping coordinates on the curve from start to end), the increment of *t* that advanced a coordinate a distance *s* along the curve was computed via a simple binary search. The algorithm accounted for the steps that crossed the join of two Bezier curves. Third, the number of coordinates that could fit onto the full curve for spacing *s* was reviewed, and if that was different from N+1, *s* was adjusted (by doubling/halving it) and the second step repeated, until the number of coordinates on the curve was N+1. The start and end of adjacent normal vectors provided four corners of a box from which pixel intensities were sampled. These methods were used to measure the variation in average signal intensity along one or more anatomically aligned curves identified in each slice image.

### Slice Alignment.

To assist the process of aligning curves from consecutive slice images, a needle was used to create visible markers in all slices corresponding to a given brain (*SI Appendix*, Fig. S11). Then, on the image of each slice three user-defined points were digitally marked on the perimeter of the needle hole, from which the parameters of a circumcircle were calculated to estimate the center of the hole. A 2D coordinate offset was then applied to the data sampled from each slice to place the alignment landmarks on an alignment axis in 3D space that we defined, by way of convention, to be parallel with the x-axis. Then, starting with the second slice image, each image was automatically rotated about the alignment axis so that the points on the curve were as close as possible to the points on the curve in the previous slice. The optimum rotations were determined by minimizing the sum of squared distances between N equally spaced locations on the curve on slice *i* and the corresponding N locations on the curve of slice *i*-1.

### Digital Unwrapping.

Digital unwrapping is the process of straightening out a curved, 3D surface into a 2D map. This process began with a set of aligned curves (*SI Appendix*, *SI Methods* and Fig. S18). We placed axis marks that defined a brain axis (white bars in *SI Appendix*, Fig. S18*A*). An unwrapping axis of zero marks was defined on the surface, by rotating a user-defined angle about the x-axis (centered on the brain axis), then locating the most distal point on each curve at this angle (blue/rainbow-colored spheres in *SI Appendix*, Fig. S18*A*). Each expression ribbon was then straightened out, keeping it fixed at its zero mark (*SI Appendix*, Fig. S18*B*). The final step was to resample the image in *SI Appendix*, Fig. S18*E* to produce an image consisting of square pixels, as shown in *SI Appendix*, Fig. S18*F*.

### Digital Reconstruction of Id2 and RZRβ Expression Patterns.

For each gene/layer, curves of the cortex were semiautomatically traced using the *Stalefish* software tools (*SI Appendix* and Movie S1). For optimal resolution, we chose 150 bins for data collection, which spanned medial to lateral. For each slice, data collection began at the most medial aspect of the medial wall (near the subiculum) and continued laterally to the rhinal fissure. While we used standard ISH, fluorescent or multicolored ISH are also easily analyzed by *Stalefish*. To align slices, we used either the *Stalefish* landmark alignment mode (where possible) or the circle-mark mode in combination with placing axis-mark data-points at the beginning and end of each brain (*SI Appendix*, *SI Methods*). To place brains of different individuals or species into a common reference frame for point-by-point comparison, we placed three landmarks at the same morphological location in each case. The first was 200 µM beyond where the boundaries of the olfactory bulb and frontal cortex first become indistinct; The second was on the slice immediately after that in which the indent of the rhinal fissure becomes indistinct and 200 µM behind the hippocampus; The third was at the most medial point of the medial wall, where the tip of the dentate gyrus creates a 0-degree horizontal line from the medial geniculate nucleus (*SI Appendix*, Fig. S16).

## Supplementary Material

Supplementary File

Supplementary File

## Data Availability

All data are available in the main text or the supplementary materials, or via the University of Sheffield's Online Research Data Archive (35). Stalefish software is available via GitHub (36).

## Change History

October 12, 2022: This article has been updated to correct a typo in an author’s name.
