# Supplementary material for: Comparing the development of cortex-wide gene expression patterns between two species in a common reference frame
Source: Proc Natl Acad Sci U S A. 2022 Oct 6;119(41):e2113896119. doi: 10.1073/pnas.2113896119 (PMC9564327; doi:10.1073/pnas.2113896119)
Supplement: Supplementary File [file pnas.2113896119.sapp.pdf]

Supplementary Materials for

Comparing the development of cortex-wide gene expression patterns between species in a common reference frame.

Sebastian S. James[2]†, Mackenzie Englund[1]†, Riley Bottom[3], Roberto Perez[3], Kathleen E. Connor[3], Kelly J. Huffman[3], Stuart P. Wilson[2]‡, Leah A. Krubitzer[1]*‡
Correspondence to: lakrubitzer@ucdavis.edu

This PDF file includes:

Figs. S1 to S19
Supplementary Text
Tables 1 to 3

Other Supplementary Materials for this manuscript include the following:

Movie S1

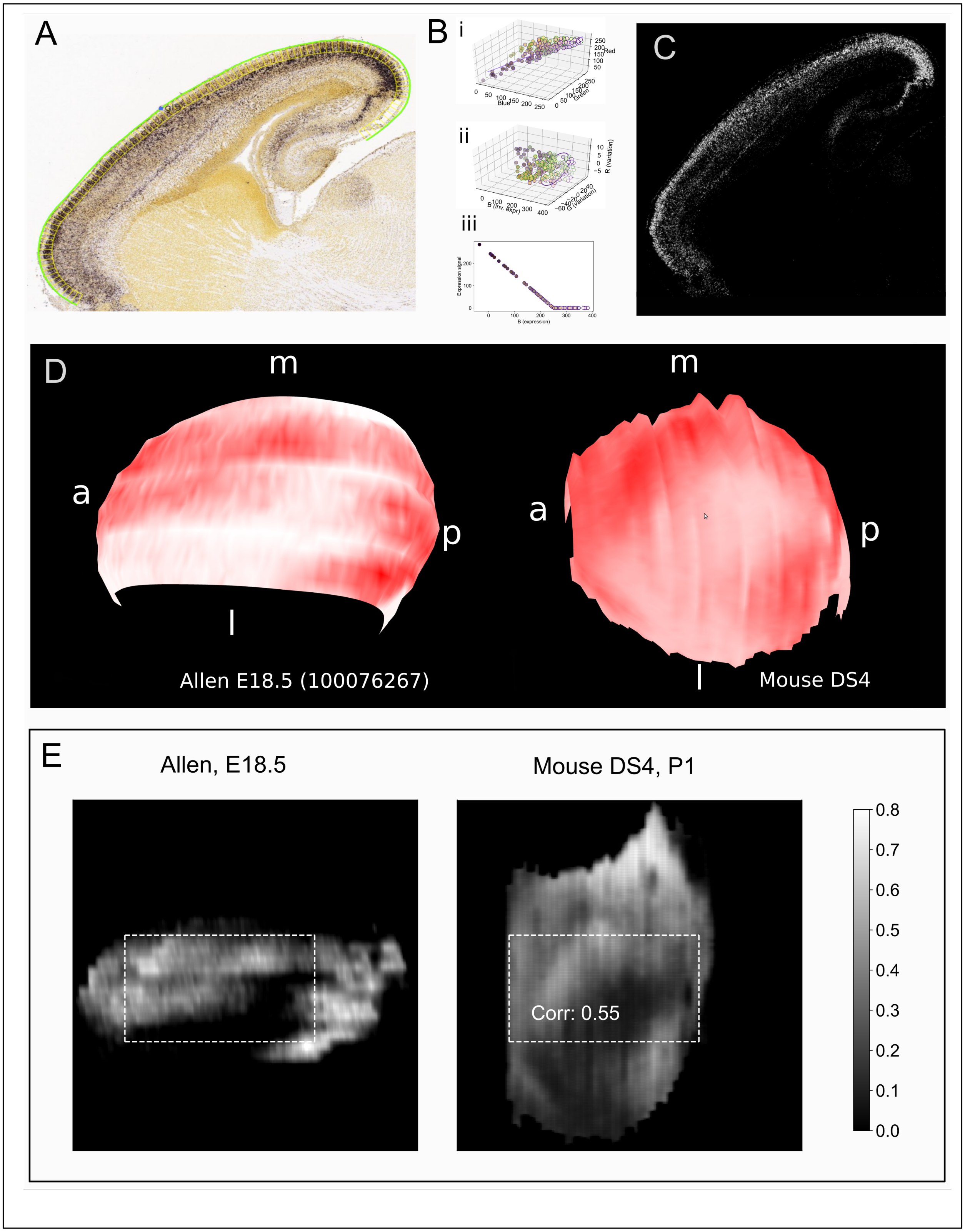
A
B i
ii
iii
C
D
m
a
p
l
Allen E18.5 (100076267)
Mouse DS4
E
Allen, E18.5
Mouse DS4, P1
Corr: 0.55
0.8
0.7
0.6
0.5
0.4
0.3
0.2
0.1
0.0

Fig. S1. ***Id2* gene expression reconstructed from Allen Developing Mouse brain slice data.** (A) Allen ISH images are colored and sagitally sliced, in contrast to our data which are monochrome stains of coronally sliced brains. In the Allen data, dark purple indicates *Id2* gene expression. (B) Extracting the signal from the image. The Allen images are provided with an associated image which shows 'expressing pixels' in a colormap but without information allowing the expression levels to be converted to a number. We set about determining a technique to convert from color values in the original images to a signal value. (i) Pixels plotted in red-green-blue 3D space. Allen-specified non-expressing pixels have a green outline, expressing pixels have a purple outline. We made a linear fit to the expressing pixels. (ii) To achieve this, we rotated the data in color space until the linear fit through the expressing pixels lies on the 'blue' axis. We allowed some variation in color about the fit by encircling the axis with an ellipse. Pixels falling within this elliptical tube were deemed to be 'expressing'. (iii) The expression level is inversely proportional to the brightness of the pixel and we set a cut-off brightness above which the expression is set to 0. (C) The resulting signal corresponding to panel A. (D) Three dimensional Layer II-III expression surfaces for the Allen data (left) and our data. Key: a: anterior, p: posterior, m: medial, l: lateral. (E) Digitally unwrapped surfaces generated from the 3D data in D. Both datasets have the same anatomically determined landmarks and the Allen data has been linearly transformed to match our data, so length scales are unified. The Allen dataset is partial (some lateral slices are missing) and so the Allen map appears 'wide and narrow'. The dotted rectangle marks the region for which both maps have data. Visual inspection of the content of the rectangles suggests that there is good correlation between the images, with a dark region of low expression from the bottom left to the middle right apparent in both maps. A Pearson correlation of the pixel values within the rectangle of 0.42 lends support for this interpretation. References: *Lein, E.S. et al. (2007) Genome-wide atlas of gene expression in the adult mouse brain, Nature 445: 168-176. doi:10.1038/nature05453*. Image used for E18.5 Id2 experiment: https://developingmouse.brain-map.org/experiment/show/100076267**.** Slice shown in panels A and C**:** http://api.brain-map.org/api/v2/image_download/101267565.

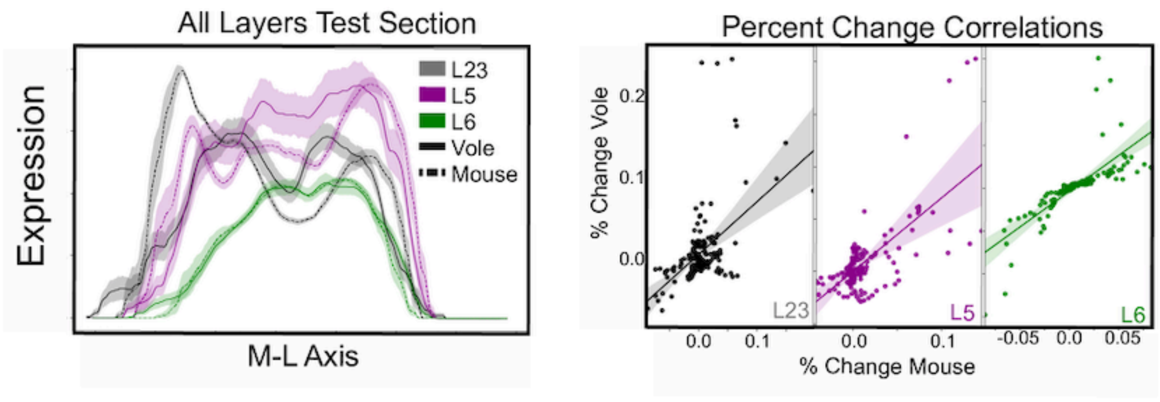

Fig. S2. **Percent Change Correlations**. Line graphs (left) show *Id2* expression profiles of layers through a single 200uM profile taken from post-transform posterior cortex: Layer II/II (L23) gray, Layer V (L5) purple, Layer VI (L6) green. Vole (solid line). Mouse (dotted line). Line graphs are presented as mean with bootstrapped 95% confidence intervals. Scatter plots (right) with line of best fit and 95% confidence intervals show correlations between expression percent change in mice (x-axis) compared to voles (y-axis) at each location along the expression profile of each layer.

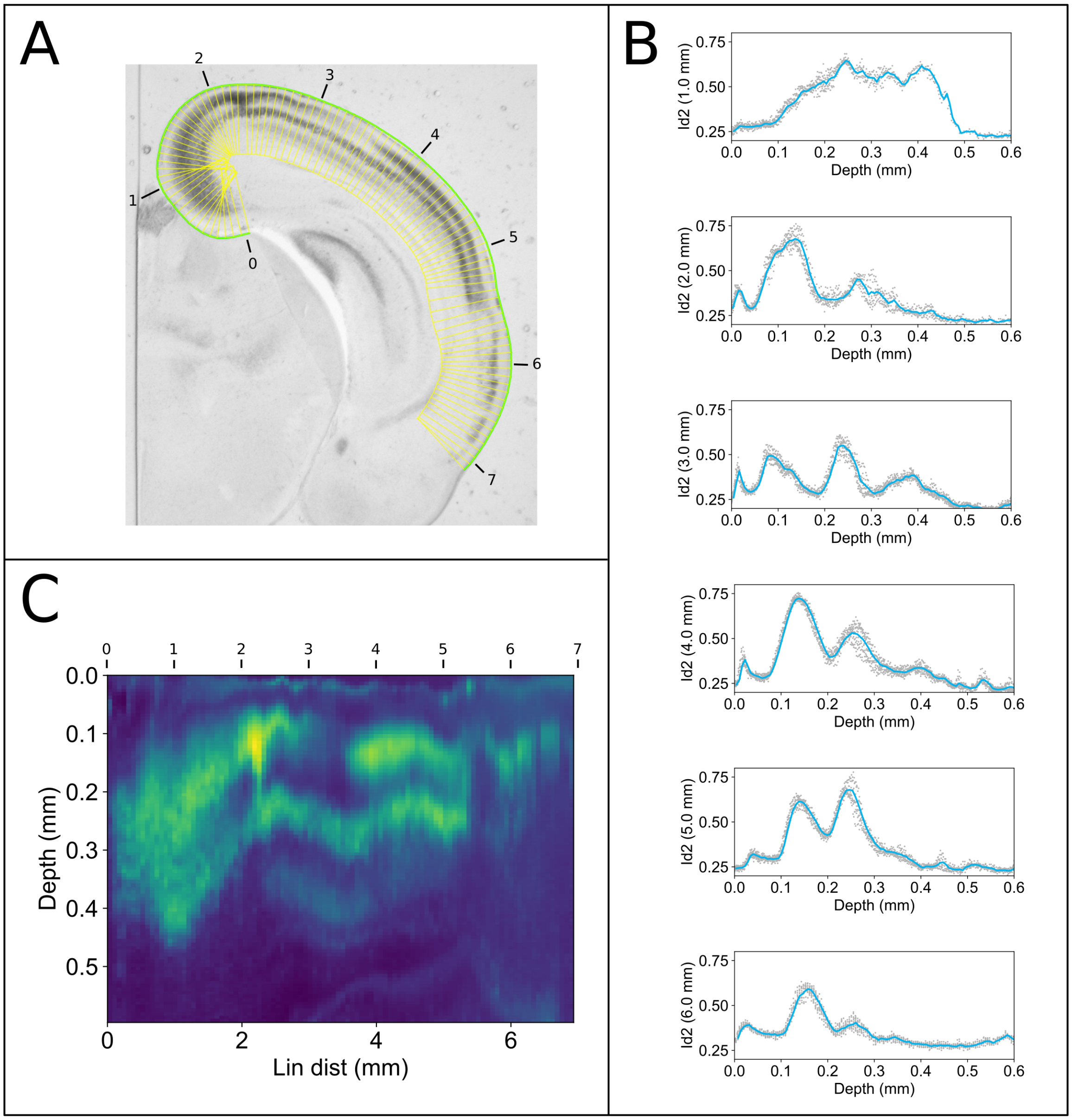

Fig. S3. Depth profiles for laminar analysis. (A) User-supplied curve is shown in green, and sampling boxes in yellow. The linear distance along the green curve is marked in black annotations (units are mm). The boxes have depth 0.6 mm. (B) Gene expression as a function of box depth for sample boxes at 1 through 6 mm of linear distance along the green line in A. Grey dots are the individual pixel values of the pixels within the sampling box, the blue line is a histogrammed mean expression (100 bins). (C) A heat map of Id2 gene expression as a function of linear distance along the green curve in A. The depth values are extracted from the blue histogram values show in B for 6 selected linear distances.

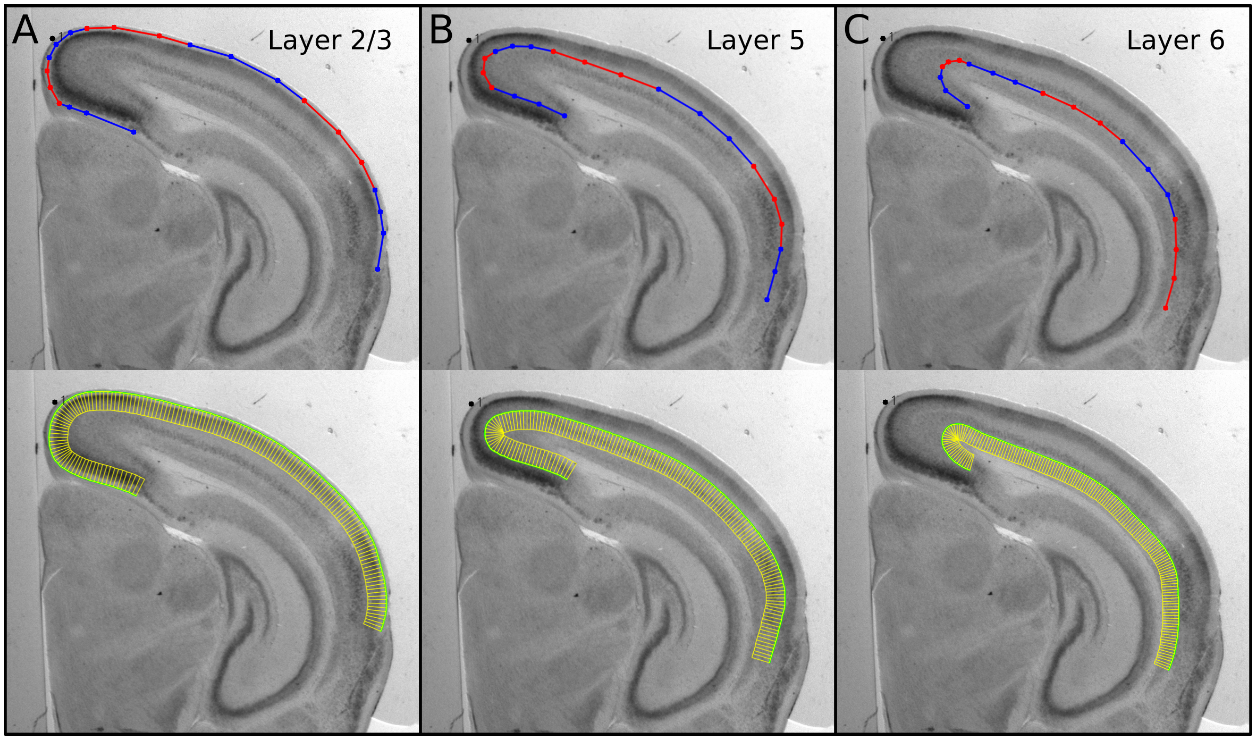

Fig. S4. Collecting layer-specific data using Stalefish We defined individual curves to collect expression data from different cortical layers. Here, we show Frame 42 of 54 for the P9 Mouse 'Pup 5' for the three curves that we defined to collect *Id2* expression from layer 2/3 (**A**), layer 5 (**B**) and layer 6 (**C**). The upper row of images show the user-defined points for the curves, the lower row indicates the curve fit in green and the sample boxes in yellow. Defining three different curves allowed us to closely follow the shape of each layer.

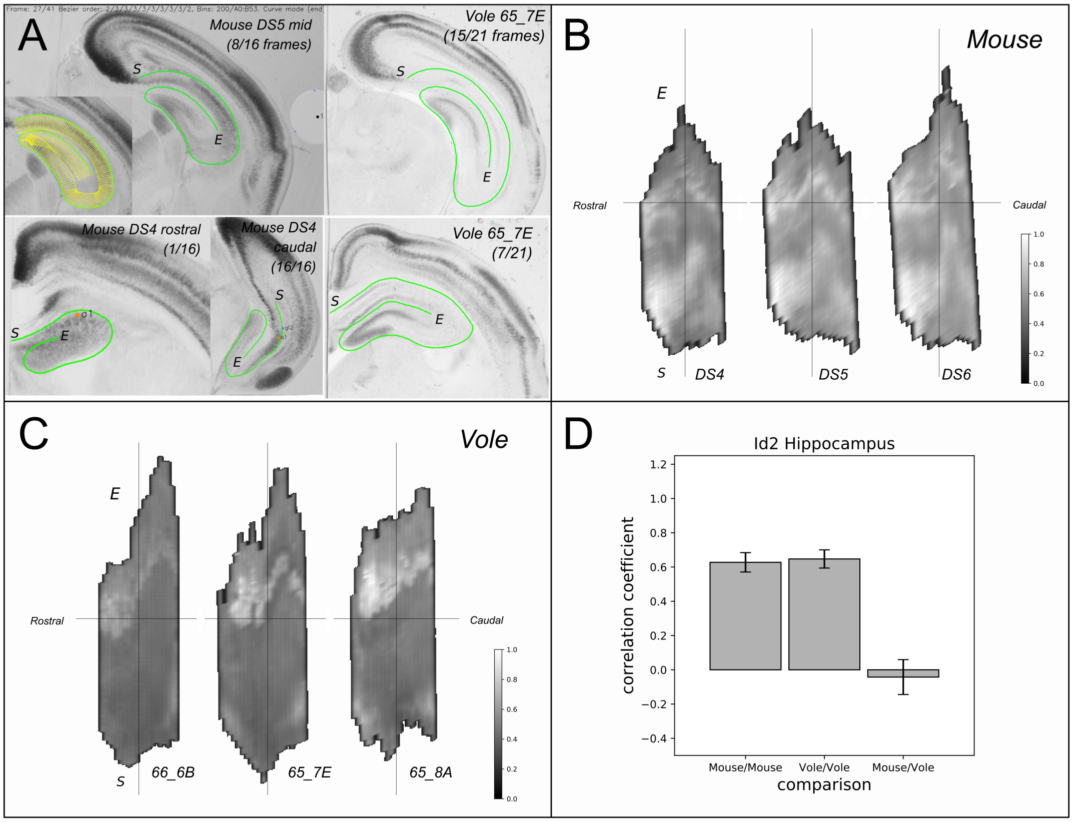

Fig. S5. **A demonstration of the use of *Stalefish* in an alternative brain structure.** Our initial motivation for developing the *Stalefish* technique was to learn about interspecies differences in gene expression in the mammalian neocortex. The ability to examine depth-based expression is ideal for studying layer-specific expression in the neocortex. However, the technique is applicable to any structure in the body and so we applied it to *Id2* gene expression in the spiral structure of the Hippocampus. (A) Spiral sampling curves allow the digital unwrapping of the hippocampus to examine and compare Id2 expression in mouse and vole. Curves were marked out in a clockwise direction; the start is marked with S and the end of each curve is marked with E. Axismarks were carefully chosen (visible the Mouse DS4 examples) to provide 'zero-angle' marks along the dorso-lateral Hippocampus. (B) Unwrapped hippocampi for three mouse brains. Each brain was marked with three landmarks, although these were based on gene expression rather than on specific anatomical features. The landmarks have allowed the mouse samples to be transformed onto the coordinate frame of one of the vole brains (66_6B). Assuming that illumination and stain response are similar for each dataset, the signals have been normalized as a group (the colorbar applies to all three maps). The Id2 expression in the dentate gyrus is visible in the top half of the maps; there is also widespread expression in the lower half of each map. (C) Similar unwrapped hippocampi for the vole, transformed onto the coordinate frame of sample 66_6B. The vole hippocampus has strong expression in the dentate gyrus, but minimal expression in the bottom half (CA1) of the maps. (D) Pearson correlation coefficients for mouse to mouse, vole to vole and mouse to vole comparisons show that the maps are well correlated within a species group, but that the expression present in the lower half of the maps for mouse destroy the correlation between species. Error bars are standard deviations for the correlations of all possible map pairs.

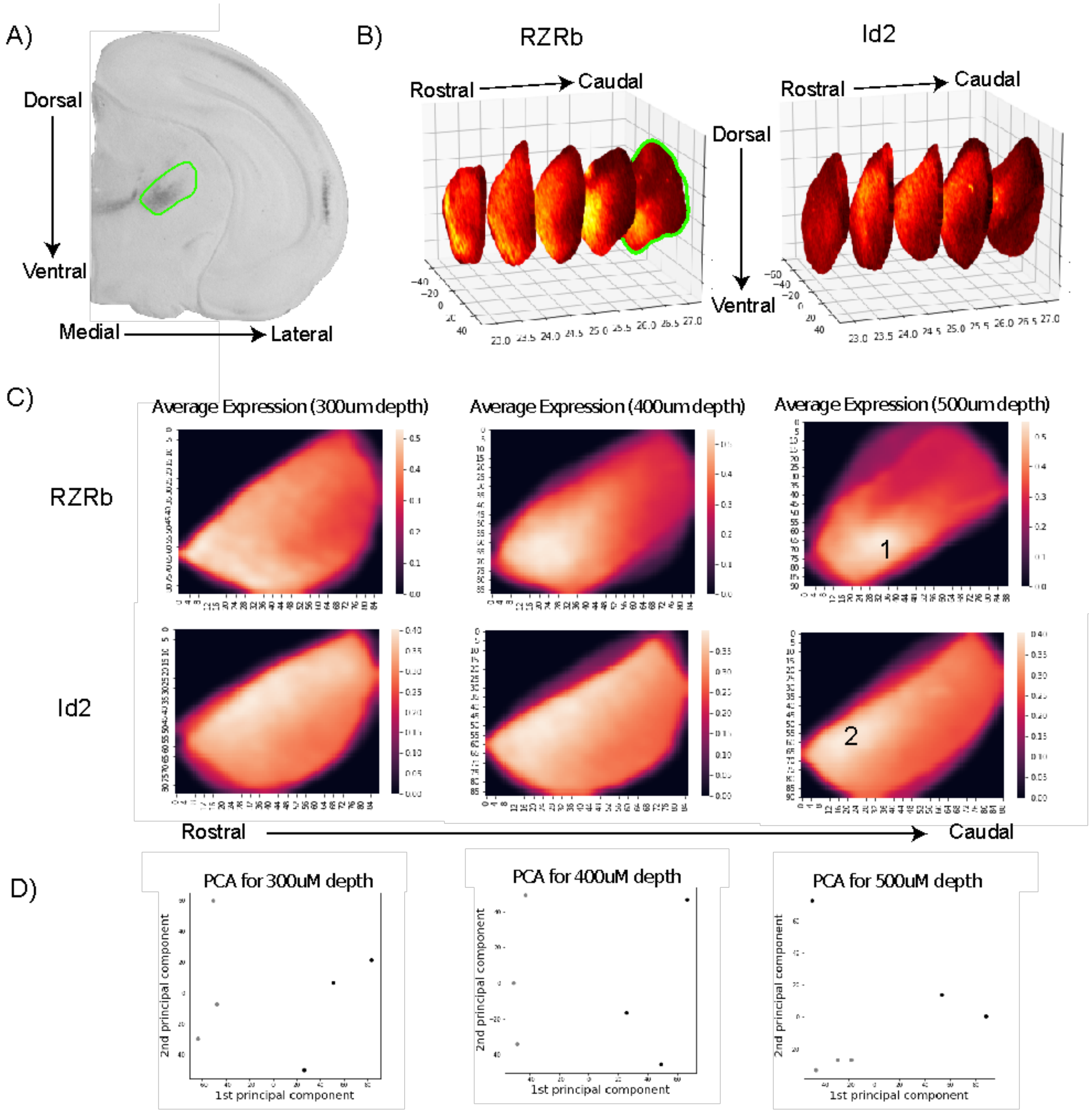

Fig. S6. Thalamic gene expression in the ventral posterior nucleus in mouse. (A) Coronal section of vole brain tissue hybridized for *RZR*β mRNA. Green circle denotes which region was analyzed using the freehand tool in *Stalefish*. (B) 3D graph representing various sections (Anterior-Posterior Plane). Green highlighted section is data retrieved from (A). Axes denote the 3 spatial dimensions of the brain. (C) Average expression maps (n=3 per slice), of serial coronal sections (akin to those shown in A, but averaged over multiple cases), for *RZR*β and *Id2*. Note how *RZR*β expression is limited to the lateral portion of VP (black number 1), while Id2 is restricted to the dorsal aspect of VP (black number 2). (D) A simple principal components analysis showing how dimensionality reduction can be used to show the relationship between the spatial expression of *Id2* (black) and *RZR*β (grey).

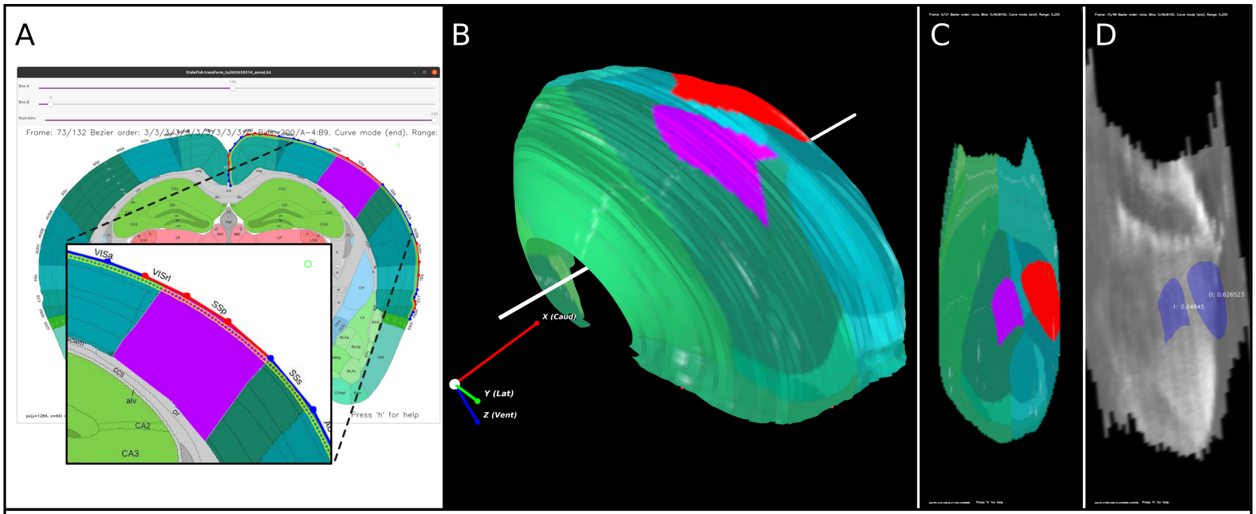

E

Id2

Condition	BC, L2/3	BC, L5	BC, L6	V1, L2/3	V1, L5	V1, L6
Vole, P1	1.4 (0.3)	1.6 (0.2)	1.8 (0.2)	1.2 (0.3)	1.4 (0.3)	1.3 (0.2)
Vole, P7	1.5 (0.1)	1.3 (0.1)	1.6 (0.2)	1.48 (0.02)	1.4 (0.1)	1.43 (0.04)
Mouse, P3	1.3 (0.2)	1.6 (0.1)	1.8 (0.2)	1.3 (0.1)	1.5 (0.2)	1.2 (0.1)
Mouse, P9	1.3 (0.1)	1.3 (0.1)	1.5 (0.2)	1.4 (0.1)	1.4 (0.1)	1.5 (0.2)

RzrB

Condition	BC	V1
Vole, P1	1.3 (0.2)	1.0 (0.1)
Vole, P7	1.19 (0.05)	1.1 (0.1)
Mouse, P3	1.16 (0.04)	0.96 (0.05)
Mouse, P9	1.1 (0.1)	1.15 (0.01)

Fig. S7. Defining putative cortical regions from the Allen P56 mouse atlas
From https://atlas.brain-map.org/atlas?atlas=602630314, we obtained anatomically annotated atlas slices of a P56 mouse brain (we used a script which queried the Allen HTTP API). **A** We loaded the images into Stalefish and defined cortical curves that would pick out the colors that define the different anatomical regions. We edited the images to modify the colors for the barrel cortex and area V1 (to purple and red, respectively) to make them easy to distinguish. The inset shows that our sample boxes were set to be very shallow, picking out the identity near the cortical surface. (B) After defining the same landmarks as for our own mouse and vole data, we were able to generate a 3D surface with anatomical region info. (C) The unwrapped cortical map. (D) Finally, we transformed our P3 and P9 mouse and P1 and P7 vole *RZR*β and *Id2* expression data onto the P56 mouse brain map shown in C. D shows
P1 Vole *Id2* expression in layer 5 (dataset 64_8A_id2_L5) with freehand-drawn sampling loops at the location of the barrel cortex and area V1, as defined by the P56 atlas. (E) The expression values found in this way are given in two tables, one for *Id2* expression, the second for *RZR*β expression. Values are given as the regional expression signal divided by the overall mean expression signal across the map (and are thus dimensionless). The numbers in brackets give the standard deviations. BC: barrel cortex; V1: primary visual cortex; L2/3: cortical layers 2 and 3 L5: cortical layer 5 L6: cortical layer 6.

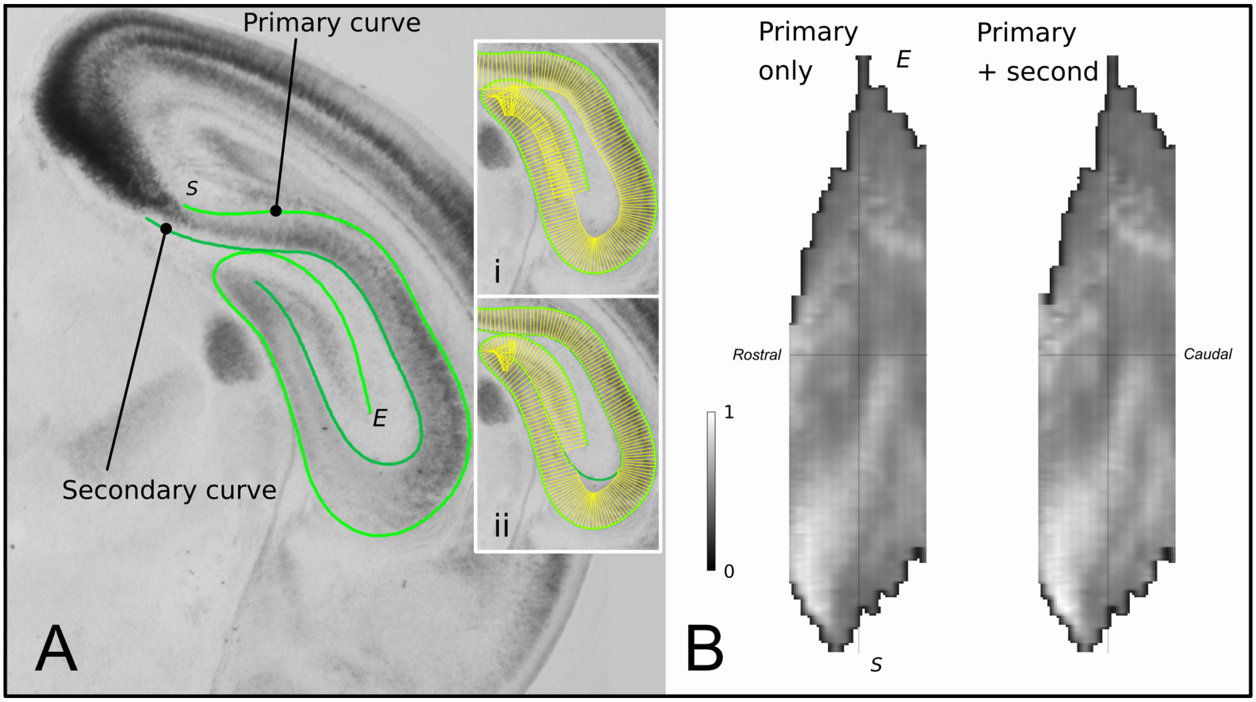

Fig. S8. Defining a secondary Curve to define sample box depth The technique of sampling from a fixed-depth box can result in sample box overlap, especially when analyzing highly curved structures. We investigated an extension to our technique, in which a second curve is defined, beyond which sample boxes will not extend. To determine whether this extension was useful, we applied it to the Hippocampus data, selecting one mouse dataset to modify. A shows the primary curve for slice 27 in the mouse DS4 data in light green, with the secondary curve shown in slightly darker green. Inset (i) shows fixed-depth sample boxes. Each sample box is created from the normal vector to the primary curve which extends 60 pixels. Inset (ii) demonstrates the use of the secondary curve. If the normal vector defining the edge of a sample box contacts the secondary curve, it ends at a depth < 60 pixels. Sample box overlap is reduced and fewer pixels are double-sampled (although some overlay remains near the sharpest part of the curve). B shows the flattened maps obtained from the DS4 slices both without (left and with (right) the secondary curve. The use of a secondary curve in this case makes very little difference to the flattened map obtained.

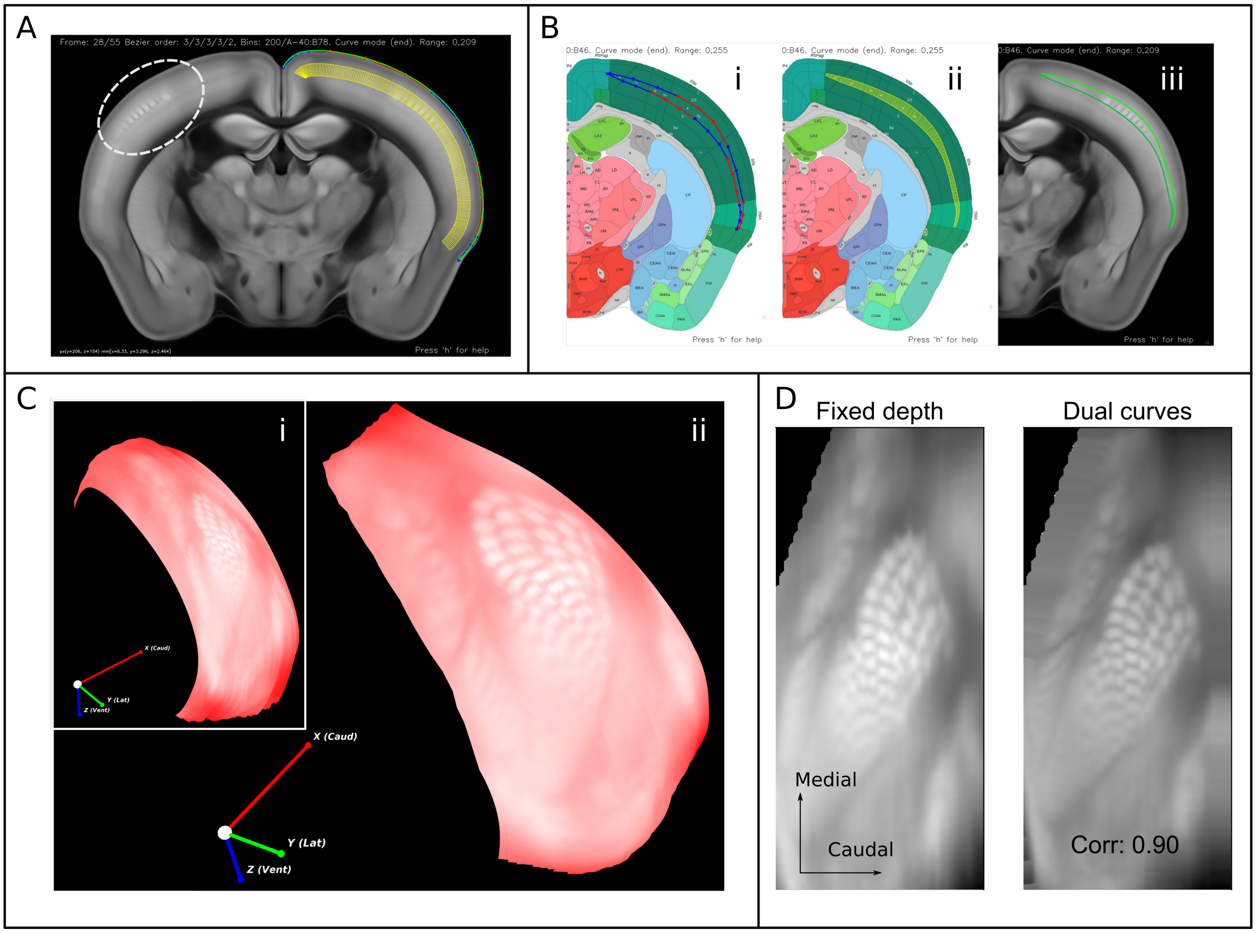

Fig. S9. Reconstructing barrels from the Allen P56 mouse atlas It is possible to obtain annotated brain atlases for mouse, such as those from the Allen Brain Institute. Here, we downloaded the 'Average Template' images for the P56 mouse coronal atlas, id 602630314 (https://atlas.brain-map.org/atlas?atlas=602630314) and loaded the images into *Stalefish*. (A) As the whisker barrels in cortical layer 4 were clearly visible (dashed ellipse) we proceeded to extract a surface from the dataset to display the map. We traced curves around the cortical surface (blue/red/green lines) and set the sampling boxes to start and end at fixed depths from the curve, covering the region in which the barrels were visible. The resulting surface, shown in (C, i) reveals an ordered whisker barrel map. (B) To compare the maps obtained by tracing the anatomy directly (as in A) and those obtained using anatomical annotations from the atlas, we loaded the annotated images into *Stalefish* and drew primary curves (i & ii) following the annotated boundaries between L2/3 and L4, with secondary curves marking the bottom of the sampling region (as in Fig. S8). These curves were then overlaid upon the 'Average Template' images (iii) and the 3D surface map was created (C ii). (D) shows the 2D maps obtained by each method, and indicates that the correlation between the images was 0.90. The 'dual curves' method extracts slightly crisper data, with both the distinct barrels that emerge with either method and also structures to the rostral side of the barrel field. This straightforward method produces a cortical map that compares well with those presented in eLife 2017;6:e18372 doi: 10.7554/eLife.18372 .

Id2

Condition	S1, L2/3	S1, L5	S1, L6	V1, L2/3	V1, L5	V1, L6
Vole, P1	1.6 (0.3)	1.6 (0.2)	1.9 (0.3)	1.4 (0.2)	1.8 (0.2)	1.9 (0.2)
Vole, P7	1.6 (0.2)	1.6 (0.2)	1.7 (0.3)	1.5 (0.2)	1.5 (0.1)	1.6 (0.1)
Mouse, P3	1.4 (0.1)	1.5 (0.2)	1.9 (0.1)	1.32 (0.04)	1.8 (0.2)	1.6 (0.2)
Mouse, P9	1.45 (0.02)	1.4 (0.1)	1.7 (0.1)	1.3 (0.2)	1.3 (0.3)	1.3 (0.3)

RzrB

Condition	S1	V1
Vole, P1	2.0 (0.2)	1.8 (0.3)
Vole, P7	1.8 (0.2)	1.6 (0.2)
Mouse, P3	2.3 (0.1)	1.7 (0.2)
Mouse, P9	1.7 (0.1)	1.6 (0.1)

Fig. S10. Using *RZR*β expression to define cortical regions. Here, we used *RZR*β expression to define sampling regions in Mouse and Vole at each age. *Id2* and *RZR*β expression was then sampled in these areas, with results given in the two tables. As in Fig. S7, values are given as the regional expression signal divided by the overall mean expression signal across the map and are dimensionless.

Supplementary Text

Introduction

This document provides an extended description of the Stalefish analysis process described in the main paper and available at https://github.com/ABRG-Models/Stalefish. Because the technique requires no special equipment, it is accessible to any lab already equipped to image histologically processed brain slices. To help other researchers create similar analyses using their own data, in the first section of this document, we present a tutorial style description of the process used to create coordinate-centered expression maps from a set of brain slices along with details of the algorithms used in the program.

The central idea of the process is to fit smooth, anatomically relevant curves to the structures visible in the 2D slices, sampling the image luminance in the region below the curve at equally spaced locations along its length. These curved sets of 'luminances below the curve' for each of the slices are then joined together so that a 3D surface is created. An algorithm makes an approximation to the 'pre-sliced' alignment of the individual slice images, by aligning each curve with respect to its neighbor (the best alignment is achieved if, during the experimental procedure a visible alignment mark is made by inserting a needle through the brain mount material). The 3D surface is then 'digitally unwrapped'. The researcher defines a 'brain axis' and an angle about this axis which forms a 'center line' through the surface. The curves are 'digitally straightened', each one being clamped to the center line. The resulting 2D map can be linearly transformed so that its coordinates match those of another brain. This is achieved by marking three anatomically identifiable locations on each brain, ideally close to the curve surfaces. The linear transform

required to transform one triplet of coordinates into the template coordinates is computed and then applied also to the luminance 'data pixel' coordinates. Finally, the map of irregularly sized, quadrilateral data pixels is resampled onto a Cartesian grid of square pixels making a regular, quantitative image which is easy to submit to standard point-by-point analysis methods.

Although we present this technique as it is applied in the main paper to in-situ hybridization (ISH) stains for the genes *Id2* and *RZRB*, it could be applied to any stain in which there is a reliable, monotonic relationship between image luminance and the value of a variable of interest. For example, this technique could be applied to cytochrome oxidase or nissl stains. Although the data presented in this work is based on monochrome ISH images, the software can also be used to interpret colored stains. As a demonstration, we include 3D reconstructions of data from the Allen Developing Mouse Brain Atlas.

In addition to a detailed description of the process in the first two sections, we provide here a step-by-step protocol for capturing expression surfaces from slice sets, and we present a number of additional analyses and visualizations to demonstrate what can be achieved once a set of expression maps have been transformed onto a common coordinate system.

<u>Sample preparation and image capture</u>

Samples are prepared in a conventional manner, with brains (Fig S11 A) fixed in gelatin-albumin (Fig S11 B) and sliced on a vibratome. The one extension to the usual method is to optionally introduce 'alignment landmarks' into the samples. This was achieved by positioning a straight, 21-gauge needle through the mold in which brains were fixed in the gelatin-albumin solution

(dissolved in 1X phosphate buffered saline and fixed with 25% glutaraldehyde). When the gelatin-albumin fixing medium solidified, the needle was removed which resulted in the circular marks (visible in Fig S11 C), which shows the resulting brain slice images. This sequence of images serves to illustrate the wide range of shapes formed by the brain as the slices are viewed in a rostral (left) to caudal (right) progression.

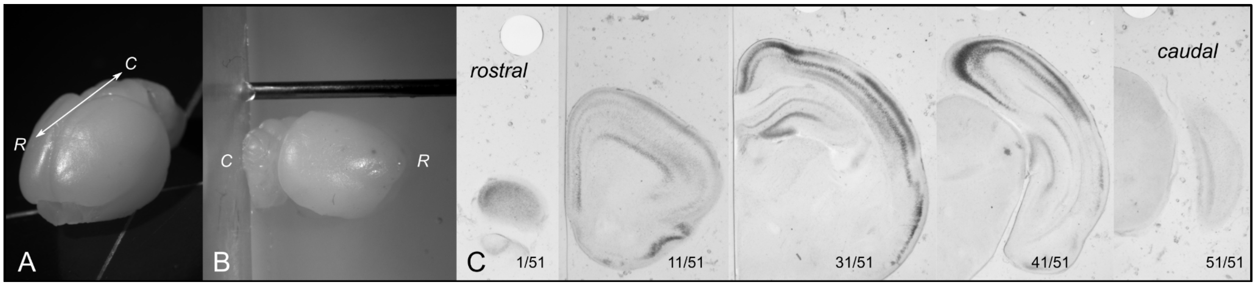

Fig. S11. (A) whole vole brain (B) The brain is positioned ready to be set in gelatin-albumin solution, with a needle in place to define the circular alignment landmarks (C) a selection of the 51 coronal sections into which another vole brain was sliced illustrating the diversity of structural shapes of the cortical region.

Bezier curves

To allow the researcher to define arbitrarily shaped, smooth curves that follow anatomical feature lines such as those shown in Fig. 1, B-D (main paper), we employed Bezier curves; a form of polynomial curve. Commonly used in drawing software, Bezier curves are typically defined by start and end locations and a series of user-editable 'control points' that lie away from the curve and determine the curvature. However, it's also possible to define a Bezier curve that best fits a sequence of points. The control points still exist but are analytically determined from the points, and thus can be assigned automatically once the user has identified several points along the edge of an anatomical feature. The number of 'user points' in the sequence determines the order of the polynomial which forms the Bezier curve. Three points gives a quartic curve; four give a cubic and N+1 points give an Nth order curve. In principle, an unlimited number of

user points could be placed along an anatomical structure and an Nth order Bezier curve fitted to them. In practice, the Bezier curve suffers from overfitting for N greater than about 5, and the curve becomes 'wobbly', passing exactly through the N+1 user points, but failing to follow the smooth curve of the structure (Fig. S12 A). However, a low order polynomial curve is limited in the complexity of the curve it can fit (Fig S12 B). The most effective curve fitting is achieved for low order polynomials applied to 'short sections' of an overall curve as in Fig. S12 C. This fails to fulfil our need to fit smooth curves to structures with complex shapes, such as the hippocampus shown in Fig S12.

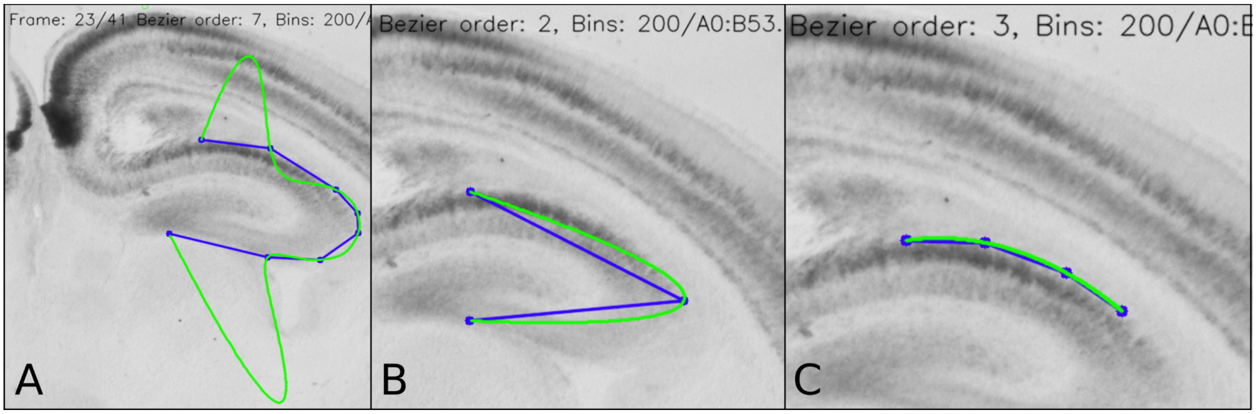

Fig. S12. (A) This 7th order Bezier curve demonstrates the problem of overfitting. The curve passes through each of the blue points perfectly, but fails to follow the real shape of the structure that the points are marking out. (B) The converse issue of underfitting, where a 2nd order curve cannot reproduce the curve around the Hippocampus. (C) A 3rd order curve fits a shorter section of the hippocampal curve.

One way to fit curves to complex structures while avoiding overfitting is to allow the user to chain several separate Bezier curves together, with each curve spanning a section of the structure that is short and 'uneventful' enough to be fit by a low-order polynomial. However, this presents the immediate problem that two curves joined together at a common point are not guaranteed to have an identical gradient at the join. As we wish to sample from boxes which extend along the

normal to the curve, this would lead to non-parallel sampling boxes at the joins. To join the Bezier sub-curves, and provide an overall smooth curve with no discontinuities in its gradient, we used a simple algorithm which modifies the control points closest to the join of the two Bezier curve segments in order that the gradient at the end of one matches the gradient at the start of the next. The algorithm is described visually in Fig. S13. In practice, the researcher adds two or three new points along the curve of the structure she is tracing (we did not fix the order of the individual 'sub-curves', allowing the user to experiment), presses a key to 'commit' the curve, then adds a few more points for another curve, repeating the process until the entire structure has been traced. It does not always produce an excellent result at the first attempt, but by cancelling and re-drawing points that express the curve of the structure, the researcher can quickly find a good fit. We have found this to be effective and straightforward enough to allow a structure to be traced across a set of 50 slices within about one hour. In future work, it may be possible to further optimize the modification of the control points at the join, to minimize the deviation of the modified curve from the user points.

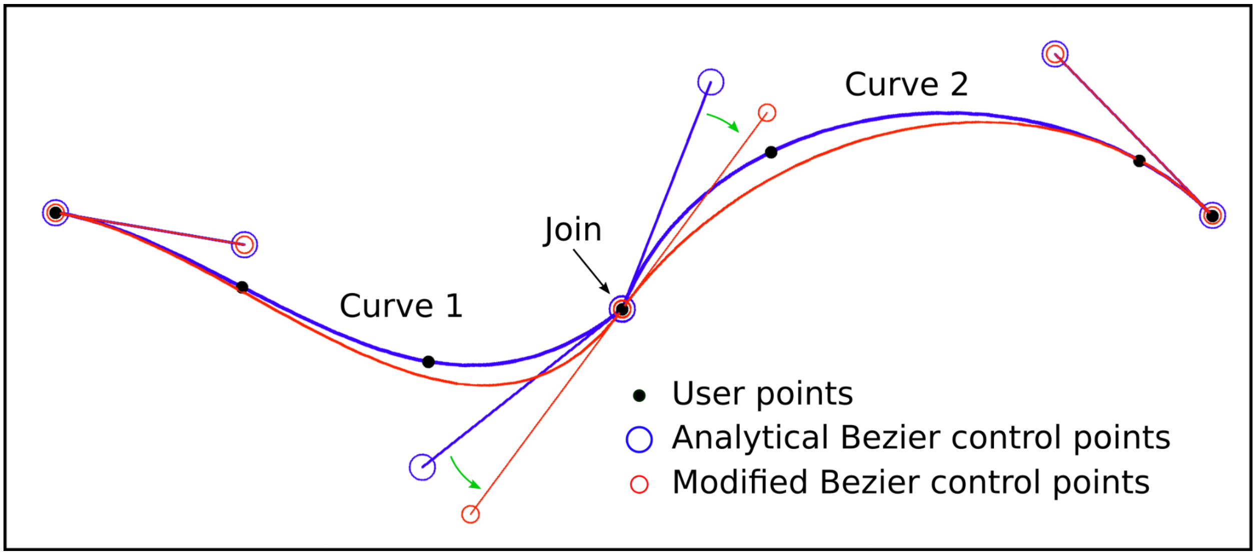

Fig. S13. Two cubic Bezier curves are shown in blue, fit to the black, user-defined points. The blue circles are the analytically determined Bezier control points that provide the best fit to the user defined points. To eliminate the discontinuity in the gradient at the join, the two closest

control points are rotated by equal and opposite angles about the join (green arrows) until they and the join lie on a straight line. The resulting, modified Bezier curve is shown in red. The modified curve no longer passes through all of the user supplied control points, but it has a smooth gradient throughout.

Sample Boxes

Once the curves have been defined, the Stalefish visualization tools can be used to display the sample boxes. N sample boxes are defined by drawing N+1 equally spaced normal vectors from the curve. To find N+1 equally spaced locations on the curve (which is made of 1 or more individual Bezier curves) we follow the following numerical procedure:

- Compute the distance from the first point on the curve to the end point (that is, the very final point of the final Bezier curve).
- Divide this by N to get a candidate spacing, *s*.
- Up to N times: advance a Euclidean distance *s* along the curve, recording the coordinate at each step. Bezier curves are parameterized with *t* in the range [0,1], mapping coordinates on the curve from its start to its end. The increment of *t* which will advance a coordinate a distance *s* along the curve is computed via a simple binary search. The algorithm takes account of steps that cross the join of two Bezier curves.
- Review the number of coordinates that could be fit onto the full curve for spacing *s*. If the number of coordinates is different from N+1, adjust *s* (by doubling/halving it) and repeat the previous step. Repeat this step until the number of coordinates on the curve is N+1.

The start and end of adjacent vectors provide four corners of a box (Fig S14 A). Controls are provided to allow the sample boxes to extend above or below the curve (Fig S14 C/D). Note that sample boxes may overlap, if the curve is sharp and the boxes extend a long distance (Fig S14

B). In future work it may be desirable to define sample boxes between two user-defined curves to avoid this problem.

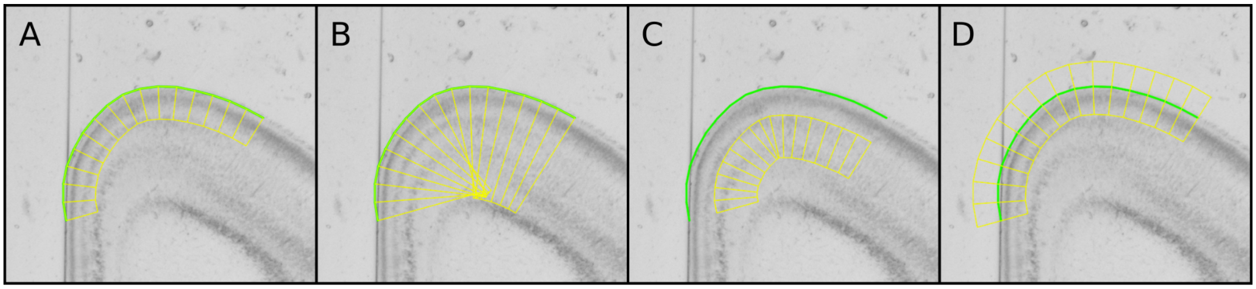

Fig. S14. (A) 16 sample boxes along a curve comprised of three cubic Bezier sections which follows the outer edge of the isocortex. (B) When the sample boxes are over-extended, they overlap, meaning that some pixels of the image will form part of multiple sample boxes. (C) It is possible to use the same curve to sample multiple regions. This example samples a deeper region than the sample boxes in A**,** making use of the same curve. (D) If required, the sample boxes may be extended above as well as below the curve.

The mean signal value in the box (and its standard deviation) is computed and stored in the Stalefish project file. Optionally, the value, coordinates and in-box depth of each pixel in each sample box can be saved into the project file.

Freehand mode

Freehand mode allows for the encircling of a region on each brain slice so that the signal encoded in pixels within the region can be saved into the HDF5 project file.

Signal recovery

The *Stalefish* technique assumes that there is some monotonic relationship between the value of a pixel in the brain slice image and a variable of interest (*Id2/RZRB* gene expression in the current study). The value of a pixel may be a simple luminance if the slice images are greyscale, or it may be that color information needs to be accounted for, such as in the Allen Developing Mouse Brain Atlas or in multiple-ISH staining techniques. We have implemented both a

luminance/greyscale color mapping and a color mapping which can be used with Allen ISH images; the choice of color mapping is selected with an entry in the project's JSON configuration file. The Allen color mapping is described in more detail in the section 'Allen mouse brain maps'.

The luminance-based mapping is a straightforward mapping of the 8-bit value of any of the color channels in the image file (any image format supported by OpenCV can be used including TIFF and PNG). The stains used here are darker where there are more mRNA molecules coding for the protein of interest, thus lower pixel luminance values correlate with higher signals. The simplest possible mapping would be to assign to the pixel value 0 the signal 1.0 and to the maximum pixel value 255 the signal 0. This would work well if the image capturing process guaranteed uniform illumination of the sample. We found that samples illuminated with a Zeiss KL1500 LCD light source and captured using a Zeiss AxioCam camera mounted to a Zeiss Stereo Discovery V12 microscope in our lab generated slight variations in luminance across the sample, which were significant enough to upset the signal extraction if some sample slices were imaged in one orientation (say, medial to the left and lateral to the right of the image) but others were imaged in the opposite orientation (lateral-medial) and then inverted in the software to match the medial-lateral slices. In these mirrored slices, the systematic overall illumination gradient was reversed making it difficult to compare the signal in adjacent slices. To counter for such inhomogeneities in the illumination, we adopted a post-processing approach. We make a copy of the image, blur it with a very wide Gaussian kernel, then subtract this from the image leaving the signal, p_s according to

$$p_s = (255 - p_i) - p_b + op_s = (255 - p_i) - p_b + op_s = (255 - p_i) - p_b + o$$

where p_i is the image pixel's 8-bit greyscale value, p_b is the pixel value from the Gaussian blurred image and o is a constant parameter (bg_blur_subtraction_offset) chosen to keep p_s in the range [0,255]. Separate windows in the Stalefish visualization can show the blurred image and the image signal. An example is shown in Fig S15.

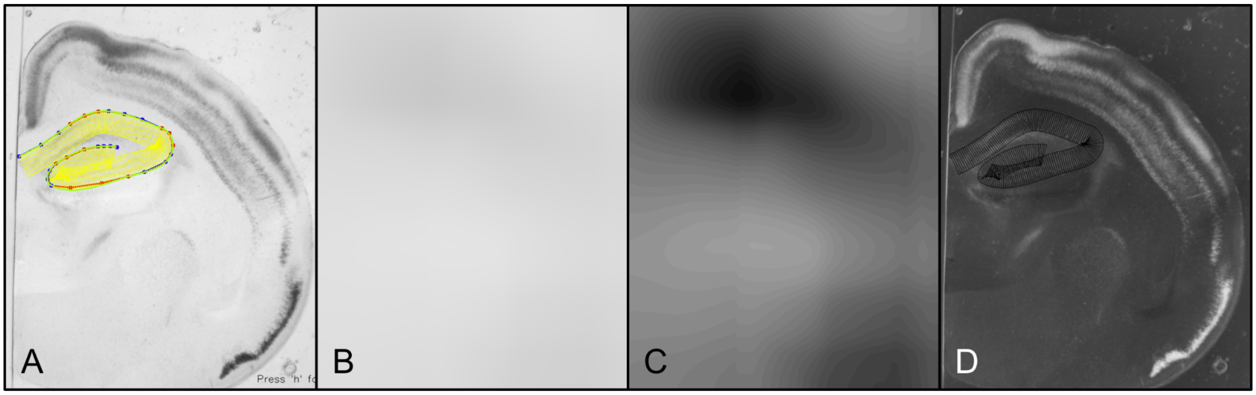

Fig. S15. (A) The main image window shows the original Id2 ISH image of slice 26 of Vole 65_7E. This is the image upon which curves, landmarks and freehand loops are drawn. (B) Gaussian blur of A with kernel width set to 1/6 of the width of the original image. Little structure is apparent because the illumination inhomogeneities in this image are small. (C) Enhanced contrast version of B indicates that subtracting the blurred image will have a small effect on true signals as well as countering any systematic illumination inhomogeneities. (D) The signal window. Signal is drawn in greyscale with higher signals towards white and so this looks like the photographic negative of the original image. The signal window can be viewed with the 'e' key in Stalefish; the blurred image with the 'r' key. Note that the sample boxes are shown on the signal window using thin black lines.

Landmarks

Landmarks are coordinates defined on the brain slice image matching either anatomical features or researcher-added alignment marks. We distinguish between landmarks which are expected to be found on every slice and those which are present on only one or a few slices. Landmarks present on every slice are used for slice alignment or for tracking structures when the 3D reconstruction has been made. Landmarks are added using *Stalefish*'s 'Landmark', 'Circlemark', 'Axismark' and 'Globalmark' modes.

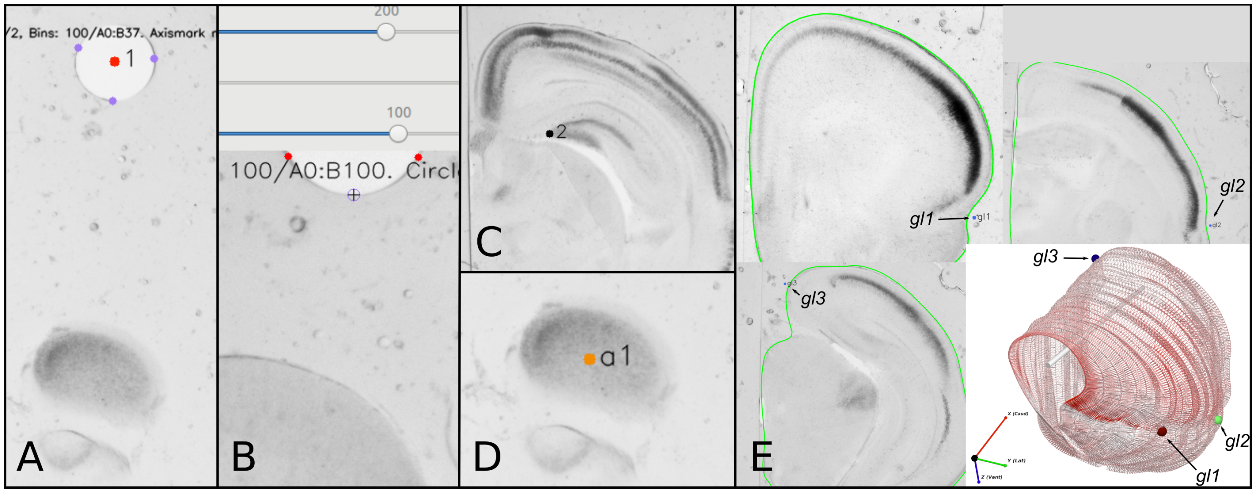

Fig. S16. Landmarks. (A) 'Circlemarks': The centre of a full circle is estimated by placing three points around its circumference and finding their circumcircle. This defines a landmark, which in this slide is numbered '1' as it is the first one. It is red because there is not a corresponding landmark '1' on every one of the other slices in the set. (B) It is possible to estimate the circle centre even if only a part of the needle-created circle is visible in the frame. (C) A regular landmark is defined by the user as a point. This landmark is marking the dentate gyrus in the hippocampus. (D) Axismarks mark the ends of the brain axis. They are marked by orange dots. (E) Three slices from a set on which are marked 3 global landmarks. The green line of the user-defined curves are shown; note that the global landmarks are defined by anatomical features but lie close to the curve in each case. The three dimensional render of the brain shows the three landmarks as spheres. These are the three landmarks which were used to make linear transformations of the digitally unwrapped map in the current study: Global landmark 1 is 200 um beyond where the olfactory bulb and frontal cortex have a less distinct boundary; Global landmark 2 is found on the slice immediately after the indent of the rhinal fissure becomes indistinct (the size and orientation of the hippocampus can be used as a guide). It is found about 200 um after a clear appearance of the hippocampus; Global landmark 3 is found at the most medial point of the medial wall, when the tip of the dentate gyrus creates a zero-degree horizontal line drawn from the medial geniculate nucleus. This mark is also associated with a change from a rounded medial wall to a more angular turn down the media aspect.

<u>Globalmarks</u>

'Globalmarks' are landmarks which are used for linear transforms. Globalmarks are stored in a data structure in the HDF5 file in the order in which they were added to the project.

Axismarks

'Axismarks' are landmarks which define a brain axis. A defined axis which passed through a brain surface is important for the digital unwrapping of the surface. The brain axis may not be aligned with any of the coordinate axes and even if it is, the user must supply a piece of information to declare which this would be as the brain may have been coronally or sagitally sliced (our convention is to say that the brain slices lie in the y-z plan and are stacked along the x axis). The user can add two coordinates to a brain slice set using 'Axismark' mode to define the endpoints of a brain axis. The use of the brain axis is described in the section 'Digital unwrapping', below.

Landmark Alignment

Once curves, or freehand regions have been drawn on all slices, we want to align adjacent brain images so that the aligned curves will form a three dimensional surface. The most reliable way to achieve alignment is to form visible markers in each slice preparation. As previously described, we used a needle to form circular marks on each slice. These circular marks were used for a 'landmark alignment' process proceeding as follows: The user marks three points on each circular landmark. The best estimate of the alignment landmark is given by the center of the circumcircle passing through the three marked points. A two-dimensional coordinate offset is applied to each slice to place the alignment landmarks in a line in 3D space that is parallel with the x-axis to form an 'alignment axis'. Then, starting with the second slice image, each slice is rotated about the alignment axis so that the points on the curve are as close as possible to the points on the curve in the previous slice. This is determined by minimizing the sum of squared distances between N equally spaced locations on the curve on slice i and the corresponding N locations on

the curve of slice *i*-1. We call this alignment technique 'landmark alignment'. It is based on the assumption that the anatomist has marked curves which correspond to the same anatomical structure on each brain slice.

We note that the best alignment accuracy using a landmark based alignment method would be to form *two* needle-formed alignment marks in the fixing medium and then perform an affine transformation of each slice image to align both alignment marks. This is left as a future enhancement to be developed in the software.

Auto Alignment

As an alternative to landmark alignment, if an existing slice set is to be analysed which does not have the necessary alignment marks, we developed an 'auto-alignment' algorithm. This uses only the two dimensional sample curves on each slice. For each slice, i, a translation, $\mathbf{r}$ and rotation, ϕ, are found which will position the curve points optimally with respect to the previous slice and a single 'target' slice. We used a Nelder-Mead optimization process *(30),* which finds a minimum for the following cost function:

$$C_i(\mathbf{r},\phi) = \frac{w_t}{N}\sum_{j=1}^{N}\|\mathbf{x}_j(\mathbf{r},\phi)-\mathbf{x}_{j,t}\|^2 + \frac{w_n}{N}\sum_{j=1}^{N}\|\mathbf{x}_j(\mathbf{r},\phi)-\mathbf{x}_{j,n}\|^2 + \frac{w_r}{1+\mathrm{e}^{-30\,|\phi-0.2|}}$$

where the first term computes the sum of squared distances between N transformed candidate points, $\boldsymbol{x}_j$ (which are evenly distributed points on the curve of slice i that have had the translation $\boldsymbol{r}$ and rotation ϕ applied) and N candidate points $\boldsymbol{x}_{j,t}$ which are evenly distributed points on the target (middle) curve in the slice set; the second term computes the sum of squared distances

between $\boldsymbol{x}_j$ and N neighboring points, $\boldsymbol{x}_{j,n}$ on slice i-1. The first term ensures that the slice positions do not 'drift' by penalizing large translations away from the centroid of the target slice. The second term ensures that each slice is closely aligned to its neighbor and the third term penalizes large rotations of any curve; it is a sigmoid curve whose parameters were set by hand to penalize rotations greater than about 0.2 radians, without affecting small rotations. w_t, w_n and w_r are weights with the values 0.01, 1 and 0.1, respectively.

Software dependencies

Stalefish was developed using the image processing library OpenCV *(31)* together with Bezier curve processing features and other supporting code from morphologica (https://github.com/ABRG-Models/morphologica). OpenGL-based visualization in the tool sfview is also provided by morphologica.

Data Analysis

This section describes how the data generated from a Stalefish project - essentially a set of mean expression values with spatial coordinates - can be rendered as a three dimensional image or converted into a two dimensional expression map. All data for a project is written into a single project file, whose format we discuss first.

Project file format

Stalefish writes data in Hierarchical Data Format, version 5 (HDF5), a global standard file format. HDF5 files can be read with a multitude of software tools and code libraries, including Python, R, MATLAB, GNU Octave, C and C++. The HDF5 project file is named to match the JSON configuration file from which the project was created. Thus, if the JSON file is called

Mouse_DS4.json, then the resulting HDF5 project file will be named Mouse_DS4.h5. The HDF5 format is standard, but the choice of variable containers in an HDF5 file is application specific. Data variable names in an HDF5 file look very much like folder paths on a computer filesystem and we refer to HDF5 variables as being contained in 'folders'. The data in a Stalefish project is divided into numbered folders; one for each slice frame; the first frame is contained in /Frame001, the second in /Frame002; etc. Each Frame folder contains a number of sub-folders containing location information, and a sub-folder which contains the extracted signal information. There is a full description of the Stalefish HDF5 format at https://github.com/ABRG-Models/Stalefish/tree/master/reading with example Python and GNU Octave code for reading (and plotting) the data available from the same location.

3D Brain

The Stalefish program allows the annotation of a set of brain slices, and saves information about the aligned data into an HDF5 file. To view and manipulate 3D renderings of the data in the HDF5 file, we wrote a simple viewer application called sfview, controlled by command line arguments. sfview can be used to visually inspect and verify the quality of the alignment of a set of slices and also to transform a set of digitally unwrapped surfaces onto a single individual example, writing out the transformed 'digital unwraps' into separate HDF5 files.

To render a gene expression surface, we must decide how to plot the mean signal value for each sample box. We have used two methods. In each method, we use the sample box vertices that lie *on* the curve. The first method uses these vertices to define a series of 'ribbons', one for each brain slice. This view, shown in Fig S17 A & B, is useful for analyzing how well the chosen

alignment algorithm has arranged the slices and how the curve shape progresses across the sample. The second method takes the on-curve sample box vertices and uses these to define a triangular mesh (Fig S17 C). This results in a smoother expression surface (Fig S17 D).

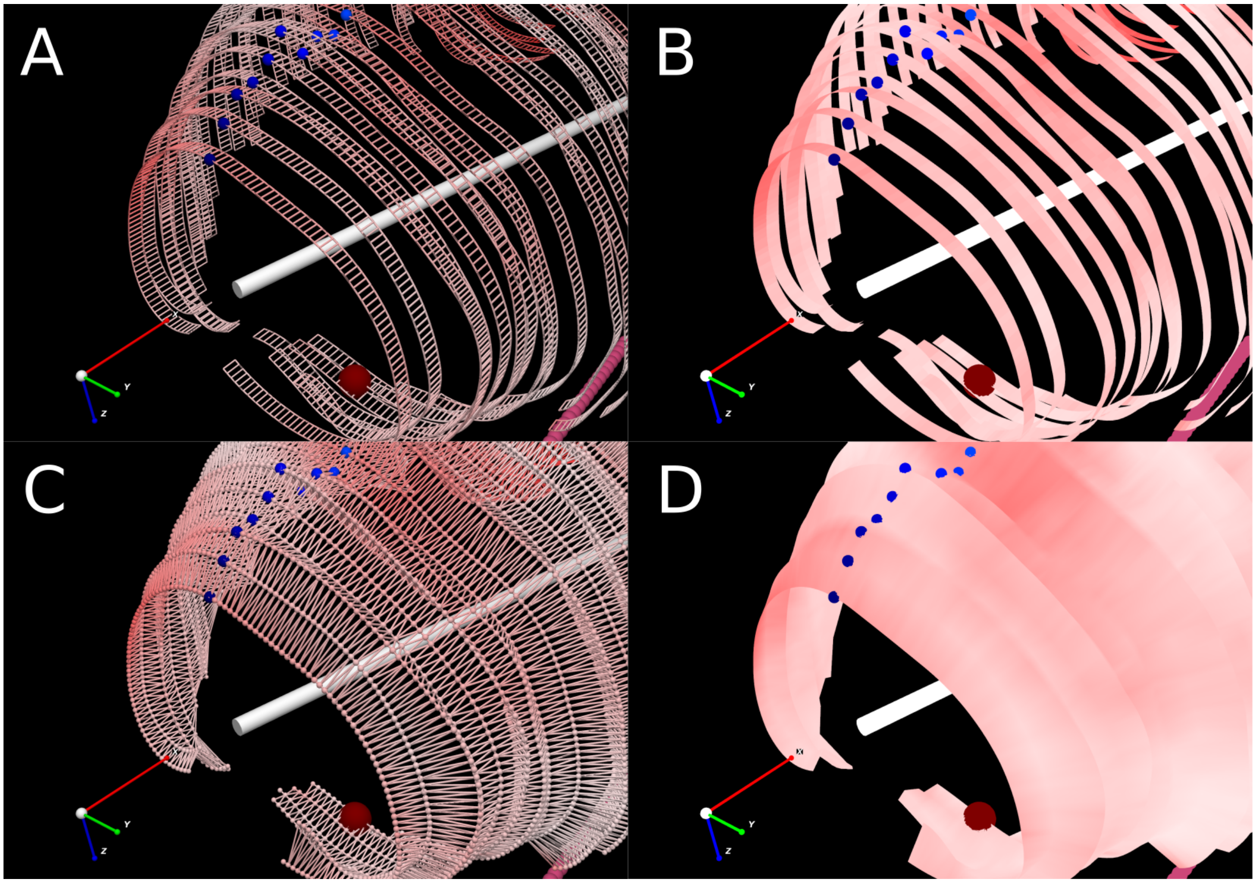

Fig. S17. Two ways to render the mean expression for the samples boxes into a three dimensional view. In each view the user-defined brain axis is shown as a white bar, the digital unwrapping 'zero marks' are shown as a row of small blue spheres and a landmark is displayed as a larger, burgundy sphere. The straight row of alignment landmarks is visible in pink at the bottom right of each panel. (A) Use two sample box vertices (each with a mean expression value) that lie on the curve, and extend along the x axis by the slice thickness to define two more vertices, forming a rectangular region of expression. The edges of each rectangle so defined are shown here to illustrate. The expression signal is shown using the color red, with the highest signal given by the most saturated red regions, but note that here, a shader that provides a diffuse lighting effect has been used and this distorts the expression colors slightly. (B) The same 'ribbon' view of the slice data, where color is defined at each vertex, but varied linearly across each rectangle (a task performed automatically by the OpenGL shader). (C) To produce a smoother surface, we use the sample box vertices on each curve to define a triangular mesh. Here, the mesh is illustrated with lines and spheres. (D) The smoothed version of C, with OpenGL performing color interpolation between the vertices as in B.

Digital unwrapping

Digital unwrapping is the process of straightening out a curved, three dimensional surface into a two dimensional map. The process begins with a set of aligned curves, as in Fig S18 A. The user provides axismarks that define a brain axis (white bar). An unwrapping axis of 'zero marks' is defined on the surface, by rotating a user-defined angle about the x-axis (centered on the brain axis), then locating the most distal point on each curve at this angle (blue/rainbow spheres in Fig S18 A). Each expression 'ribbon' is now straightened out, holding it fixed at its zero mark (Fig S18 B). In Fig S17 C, the straightened ribbons have been inverted and a further rotation shown in Fig S18 D shows that the data begin to resemble the two dimensional map in Fig S18 E, in which the zero marks have been arranged to lie on a straight line, which means that there are now no gaps between the ribbons. Note that the quadrilaterals which make up the 'pixels' in Fig S18 E are not of even size; those in the shorter ribbons are smaller than those in the longer ribbons (because in this example, there are the same number of sample boxes on each curve/ribbon). Furthermore, although this particular map has not been transformed; it is possible that a transformation may be applied to the map in Fig S18 E, transforming rectangular pixels into general quadrilaterals prior to resampling. The final step is to resample the image in Fig S18 E to produce an image consisting of square pixels, as shown in Fig S18 F.

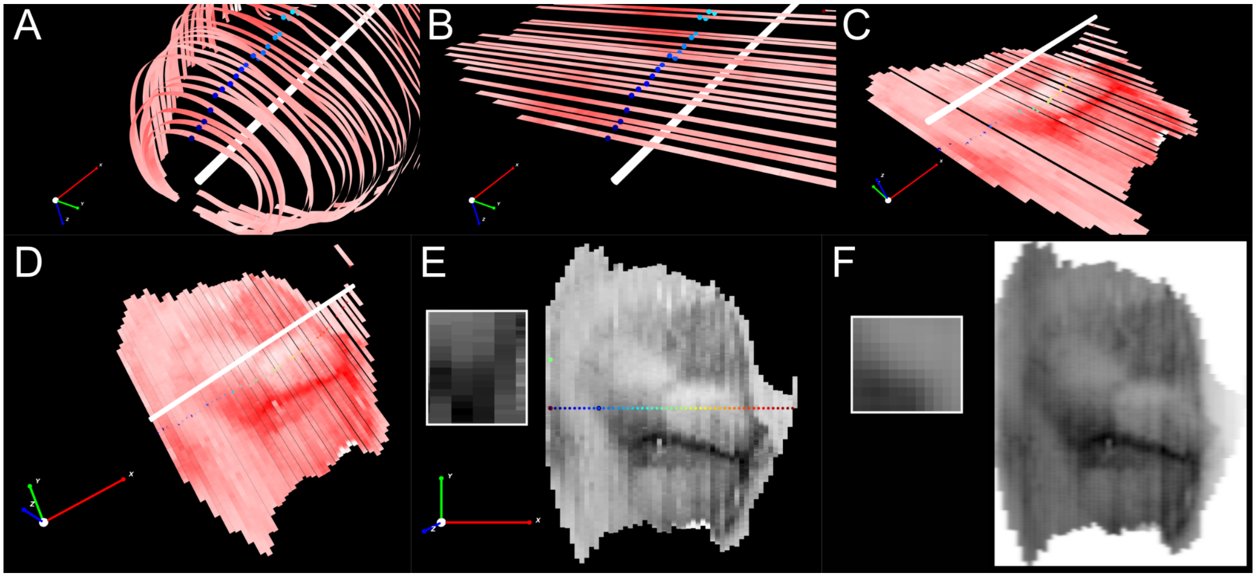

Fig. S18. The digital unwrapping process. (A) To illustrate the process, we start with a Vole brain with Id2 expression shown as 'ribbons' which follow the curves defined in Stalefish. The brain axis is visible as a white bar and at a fixed angle about the x axis, a series of 'zero marks' are shown on the ribbons as rainbow colored spheres. (B) The ribbons are straightened out using the zero marks as fixed points. The 3D view in A and B is identical; the brain axis and zero marks are unmoved. (C) The view is zoomed out and inverted with respect to panel B (compare the xyz coordinate arrows) (D) Further rotation of the 3D view. The gene expression pattern is now visible. (E) The unwrapped ribbons are now aligned by taking the zero marks and arranging them along a straight line. Note that this image still consists of quadrilaterals of varying size (inset). There are as many quadrilaterals in the short end ribbons as in the long central ribbons. (F) The image is resampled using a sum of Gaussians method to produce the final image, whose pixels are now square (inset).

The resample algorithm finds a signal value, p_k, for each square pixel in a resampled grid (Fig. S18 F). p_k is a sum determined from the contributions of M quadrilaterals indexed by j, in each of N ribbons according to a 2D elliptical Gaussian distribution centered on each quadrilateral. The parameters of each elliptical Gaussian are determined by the shape of the quadrilaterals. This can be expressed as

$$p_k = \sum_{i}^{N} \sum_{j}^{M} s_j \exp(-a(x_k - x_j)^2 + 2b(x_k - x_j)(y_k - y_j) + c(y_k - y_j)^2)$$

where s_j is the signal of quadrilateral j, (x_k, y_k) are the coordinates of the square pixel; (x_j, y_j) are the coordinates of quadrilateral and a, b and c are given by

$$a = \frac{\cos^2\phi_j}{2{\sigma_{j,x}}^2} + \frac{\sin^2\phi_j}{2\sigma_{j,y}} \qquad b = \frac{\sin 2\phi_j}{4{\sigma_{j,x}}^2} + \frac{\sin 2\phi_j}{4{\sigma_{j,y}}^2} \qquad c = \frac{\sin^2\phi_j}{2{\sigma_{j,x}}^2} + \frac{\cos^2\phi_j}{2{\sigma_{j,y}}^2}$$

where $\sigma_{j,x}$ and $\sigma_{j,y}$ are the parameters of an ellipse rotated by the angle ϕ_j. We used 3 corner coordinates of the quadrilateral ($\mathbf{c}_1$, $\mathbf{c}_2$ and $\mathbf{c}_3$) to determine these parameters. Suitably chosen, these give two basis vectors, $\boldsymbol{x}' = \mathbf{c}_3 - \mathbf{c}_2$ and $\boldsymbol{y}' = \mathbf{c}_1 - \mathbf{c}_2$ for the quad which define the major and minor axes of the ellipse:

$$\sigma_{j,x} = \frac{|\mathbf{x}'|}{2}; \quad \sigma_{j,y} = \frac{|\mathbf{y}'|}{2}$$

The rotation of the ellipse, ϕ_j , is defined as the angle which $\boldsymbol{x}'$ makes with respect to the x axis, i.e.

$$\phi_j = \arctan\frac{\mathbf{x}'_y}{\mathbf{x}'_x}\phi_j = \arctan\frac{\mathbf{x}'_y}{\mathbf{x}'_x}\phi_j = \arctan\frac{\mathbf{x}'_y}{\mathbf{x}'_x}$$

Min/max mode

Stalefish allows the user to specify a minimum and maximum signal location on any given frame. These values are recorded in the HDF5 project file as /FrameN/signal/postproc/manual_min_signal and /FrameN/signal/postproc/manual_max_signal (where N is the frame number). These values can then be used to scale the frame's signal.

Protocol for processing images to generate 3D and 2D surface expression maps

1. Create a text file with a .json suffix and populate it with the mandatory elements given in Table 1 and with reference to the example in Fig S19.

2. Launch Stalefish with the path to the .json file as a single argument. It will load the images and present the first one to the user in two windows, one a 'working' window and a second which displays the mRNA signal to the user (after subtracting the blurred background).

3. Cycle the input mode to 'Circlemark' mode (see Table 2 for a list of Stalefish functions). This allows the location of the alignment needle mark to be set for each slice. Place three marks around the circular boundary of the needle hole allowing the program to mark the center of the hole. Repeat for each slice in the set.

4. Cycle the input mode to 'Axismark' mode. This is used to define a central axis through the brain samples. Mark exactly two axis marks in the entire slice set.

5. Cycle to 'Curve' mode. Using the mouse, place 3 or 4 points to define a part of the curve on the slice. Press space to commit a curve portion; it will turn red or blue. Cancel points and replace them as necessary until the curve portion follows the anatomical structure satisfactorily. Define 3 or 4 more points along the curve and press space to commit a new curve portion. Continue until a full curve has been defined for the structure of interest. Repeat for all brain slices.

6. Cycle to 'Global landmark' mode. Define exactly three global anatomic landmarks across all of the slices in the brain. Each landmark should ideally be relatively close to the curve.

7. Use the save function to write the data to an HDF5 file (the structure of the data content in this file is described separately). Exit Stalefish.

8. If analyzing a single brain, open the HDF5 file using the sfview program to verify that the slice alignment and 2D map unwrapping was successful. Use the -m1 argument to view the 2D unwrapped map. For example, if the json file was named brain1.json, the HDF5 file will have been named brain1.h5 and the correct sfview command would be `./build/src/sfview brain1.h5 -m1`

9. If analyzing several brains, then follow steps 1 to 7 to define curves and global landmarks on each brain. The brain maps can be transformed onto the same coordinate axes using sfview's -T argument, which computes transformations based on the 3 global landmark coordinates provided on each brain. For three brains, an example sfview command is:

./build/src/sfview brain1.h5 brain2.h5 brain3.h5 -m1 -T

In this case, brain2 and brain3 would be linearly transformed to match brain1 (which would not be transformed). To transform to match brain2, brain2.h5 would be given as the first argument. sfview will write out the transformed and resampled data into separate .h5 files with a naming scheme showing which 2D brain map is stored and from which brain its transformation was computed ('Transformation From'). The example above would result in these files:

brain1.TF.brain1.h5
brain2.TF.brain1.h5
brain3.TF.brain1.h5

**JSON element name**	**Type**	**Description**
**Mandatory elements**		
thickness	real number	The thickness, in mm, of the brain slices (assumed to be same for each slice).
pixels_per_mm	integer	Conversion factor from pixels in the slice images to mm (slice image pixels are assumed to be square).

map_align_angle	real	Angle in radians about the brain axis defining a center line about which the 2D brain map is unwrapped from the 3D brain.
slices	array of JSON objects	Each member of this json array is a json object containing: "filename" (string), the filepath (relative or absolute) to a brain slice image file, and, "x" (real number), the position along the x axis at which the brain slice is located
**Optional elements**		
scaleFactor	real	A factor to scale the images by as they are loaded into the program. Can help to display high resolution images on a lower resolution computer monitor. Note that the data signal is collected from the scaled image, not from the original image. For best results omit scaleFactor, or set it to 1.
bg_blur_screen_proportion	real	bg_blur_screen_proportion is multiplied by the width of the image in pixels to get a sigma for the Gaussian which is used to blur the image to subtract the background.
bg_blur_subtraction_offset	real	signal_img = 255 - (original_img + (bg_blur_subtraction_offset - blurred_bg)). Thus, possible range for bg_blur_subtraction_offset is 0 to 255.
save_per_pixel_data	Boolean	If true, then save out the signal values and coordinates of every individual pixel in each box and freehand loop. Not required to make surface maps and can lead to a very large HDF5 data file. Default is false.
save_auto_align_data	Boolean	If false, then don't write coordinates in the autoaligned frame of reference into the HDF5 data file. Default is true.
save_landmark_align_data	Boolean	If false, then don't write coordinates in the landmark aligned frame of reference into the HDF5 data file. Default is true.
rotate_landmark_one	Boolean	If true, and there is >1 landmark per slice, apply the 'rotate slices about landmark 1' alignment procedure anyway. Normally, the rotational

		alignment is applied by default only if there is EXACTLY 1 landmark per slice. Default is false.
rotate_align_landmarks	Boolean	If true, then in 'rotate about landmark 1 mode' align the other landmarks, instead of the curves. Default is false.
colourmodel	Text	If "allen" then apply Allen Developing Mouse Brain color mapping to convert color to scalar signal. If "raw_colour" then record the RGB color values in each pixel, rather than a scalar signal. If "sfview" then expect images in .h5 format. Otherwise, apply luminance/greyscale mapping with background offset to obtain a scalar signal.
colour_trans	Array (real)	1x3 array specifying a color translation for the Allen color model.
colour_rot	Array (real)	1x9 array specifying a color rotation for the Allen color model.
ellip_axes	Array (real)	1x2 array specifying the dimensions of the ellipse used in the Allen color model. This is a red-green ellipse (after colour_trans and colour_rot have been applied to the data) for the "elliptical tube of expressing colors"
luminosity_factor		The slope of the linear luminosity vs signal fit.
luminosity_cutoff		Defines the luminosity at which the signal cuts off to zero.

Table 1: *mandatory and optional parameters which should be written into a Stalefish project's JSON configuration file.*

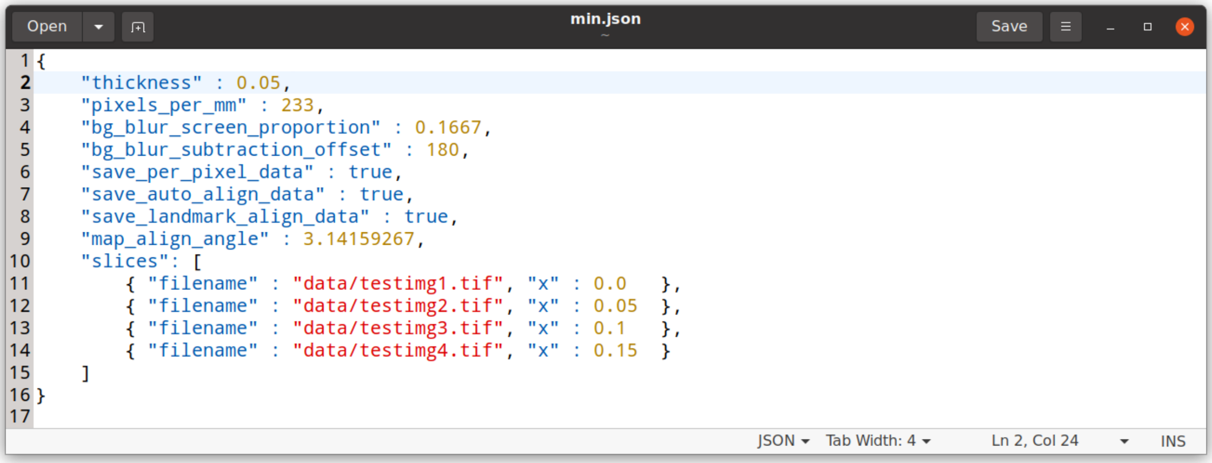


```json
{
    "thickness" : 0.05,
    "pixels_per_mm" : 233,
    "bg_blur_screen_proportion" : 0.1667,
    "bg_blur_subtraction_offset" : 180,
    "save_per_pixel_data" : true,
    "save_auto_align_data" : true,
    "save_landmark_align_data" : true,
    "map_align_angle" : 3.14159267,
    "slices": [
        { "filename" : "data/testimg1.tif", "x" : 0.0   },
        { "filename" : "data/testimg2.tif", "x" : 0.05  },
        { "filename" : "data/testimg3.tif", "x" : 0.1   },
        { "filename" : "data/testimg4.tif", "x" : 0.15  }
    ]
}
```

Fig S19. An example Stalefish JSON configuration file. This example contains the mandatory elements, plus a few of the optional elements. The project contains four slice images. Images may be given in TIFF, PNG or JPEG formats, or they may be the 2D maps generated as HDF5 files by sfview with the -m1 option.

**Function**	**Key**	**Description**
Box A	user interface slider	'Sample box position A'. Change the position of the start of the sample boxes
Box B	slider	'Sample box position B'. Change the position of the end of the sample boxes
Num bins	slider	Change the number of sampling bins on the curve (range: 2-200)
Toggle Bezier control points	1	Toggles the visibility of the Bezier control points for the curves
Toggle user points	2	Hides/shows the user-supplied control points
Toggle fit line	3	Hides/shows the green line of best fit to the user-supplied control points
Toggle the bins	4	Hides/shows the yellow sample bins
Toggle text	5	Hides/shows text annotations
Move to next curve	Space	Commits the green 'pending' user points to be a Bezier curve; new user points will become part of the next curve.

Cancel	c	Cancel the last point or freehand region, depending on context. If in *Curve mode* it cancels the last user-supplied curve point; if in *Globalmark mode* then the last global landmark is cancelled.
Delete all curves	C	Clear all user-supplied points from the current project
Update fit	f	Recompute the Bezier fit on the current frame
Update fit (all frames)	F	Recompute the Bezier fit on the all frames
Copy bin params	B	Copy the sample bin params to all other frames
Copy objects from previous frame	P	Copy curves and freehand loops from the previous frame to the current frame
Copy objects from next frame	N	Copy curves and freehand loops from the next frame to the current frame
Write file/Save	w	Write the project to an HDF5 file
Cycle mode	o	Cycles the input mode between: 1) Curve mode; 2) Freehand mode; 3) Landmark mode; 4) Globalmark mode; 5) Circlemark mode; 6) Axismark mode; 7) Min/max mode; 8) Curve2 mode (secondary curve)
Curve mode: start/end	s	When in curve mode, this switches the input to add (or cancel) user points to either the start or the end of the curve. Adding at the end is default.
Export user points to tmp	k	Export data to /tmp/landmarks.h5, /tmp/curves.h5 and /tmp/freehand.h5
Export landmarks etc to tmp	p	Export mode-specific data to /tmp
Export curves/loops to tmp for single frame	[	Export mode-specific data to /tmp for the current frame. Mode specific data can then be imported to another frame in the same, or a different project.
Import landmarks	l	Import landmarks from /tmp/landmarks.h5
Import curve points	i	Import curve points from /tmp/curves.h5. If curves saved in /tmp/curves.h5 is from single frame

		(saved with '['), then they will be imported into the current frame.
Import freehand	j	Import freehand loops from /tmp/freehand.h5. If loops saved in /tmp/freehand.h5 is from single frame (saved with '['), then they will be imported into the current frame.
Next frame	n	Move to the next frame in the brain slice set
Back to previous frame	b	Move to the previous frame in the set
Move frame backwards	8	Reorganize frames by moving the current frame backwards
Move frame forwards	9	Reorganize frames by moving the current frame forwards in the set
Mirror frame	m	Mirror the image (left-right)
Toggle blur window	r	Toggle the window that shows the Gaussian-blurred background
Toggle signal window	E	Toggle the window that shows the final signal
Toggle image data	q	Toggles the image in the main window, allowing the curves and landmarks to be viewed without the background data.
Load example data	e	Load the built-in example project (Vole, Id2)
Help	h	Displays a summary of the key functions on screen
Exit	x	Exit the program (press twice to confirm)

***Table 2:** Stalefish functions (accessed by key-press)*

**Function**	**Key**	**Description**
Help	h	Output a helpful key summary to stdout
Exit	x	Exit sfview
Scene lock	l	Toggle the scene lock to prevent mouse movements

		from changing the view
Mouse rotate mode	t	Toggle the axes about which the scene is rotated for mouse movements
Coord arrows	c	Show/hide the small coordinate arrows
Snapshot	s	Take a snapshot, which will be saved as picture.png
Reset view	a	Reset to the default viewpoint
Reduce field of view	o	Reduce the field of view of the virtual camera
Increase field of view	p	Increase the field of view of the camera
Save view	z	Saves the current view location to a temporary file, which will be restored on future runs of the program
Reduce the near cutoff plane	u	Reduce the plane beyond which objects are rendered
Increase near cutoff plane	i	
Select model	0-9	When sfview is showing multiple expression surfaces, they can be individually selected with the number keys
Decrease opacity	Left	Decrease the alpha channel value for the selected model, making it more transparent
Increase opacity	Right	Increase the alpha channel value for the selected model, making it more opaque
Toggle landmarks	f	Show/hide the landmark locations
Toggle zero angle marks	g	Show/hide the zero angle marks which are used to digitally unwrap a 3D surface into a 2D map
Toggle brain axis	d	Show/hide the user-defined brain axis
Toggle 2D map	j	Show/hide the unwrapped 2D map
Toggle 3D map	k	Show hide the 3D expression surface

Table 3: *sfview functions*

Considerations

The reconstruction of 3D expression data for the Allen Developing Mouse Brain Atlas was achieved by registering 2D brain slice images to their neighbours using a mutual information metric (Mattes et al. 2001), which compares each pixel in two neighbouring slices and finds a translation and rotation to apply to one slice such that the difference between the two images is minimised. To overcome a problem whereby the registration along the stack can 'drift', the slices were also registered to a reference mouse brain obtained by serial two-photon tomography (Ragan et al. 2012). Once aligned, the expression levels from the 2D images were resampled to produce a 3D cloud of expression levels. This 3D cloud can then be analysed and expression levels within different brain regions can be computed, with the help of the reference brain. The present technique is distinct in that rather than using the entire brain image to register neighbouring slices, it instead allows the researcher to trace out a structure of interest on each slice and then uses the shape of these structures to perform the alignment. By allowing the anatomist to trace the curve of a brain structure on each slice, this approach naturally yields 3D manifolds (or surfaces) of expression, rather than a 3D point cloud. In this way, our techniques make it possible to analyse the maps of expression to identify common features between brains.

Stalefish helper programs

There are two helper functions for Stalefish. *sfgetjson* extracts the JSON configuration from a Stalefish HDF5 project. *sfmakejson* will generate a Stalefish JSON project file from a collection of image files.

Future development of *Stalefish*

Stalefish is a simple tool, implementing the well-defined and limited set of algorithms described in this work. We followed the design philosophy of doing a simple thing and doing it well. As such, we hope that significant future development of the software will not be necessary. However, some improvement may be desirable in the algorithm which joins separate Bezier curves together, with a more sophisticated optimization applied to the way that the algorithm adjusts the adjoining curves' control points.

The data generated by *Stalefish* is intended to be rendered in any tool of the researcher's choice. One possibility would be to use the recently developed BrainRender *(33),* though we have not yet investigated any changes that may be necessary to make this work.

An aspect of the *technique* that warrants future development is the definition of anatomical landmarks and the way that three dimensional surfaces are digitally unwrapped. We have one feature in particular in mind; 'manual unwrapping'. Here, rather than using the angle about the brain axis to define the 'zero marks' about which the surface is unwrapped, it might be possible to use manually placed landmarks on each brain slice. This might work for the Hippocampal data in Fig. S5 for which the dentate expression on each slice could be used to define the unwrap 'zero-mark'.

Additional References:

T. Ragan, *et al.*, Serial two-photon tomography for automated ex vivo mouse brain imaging. *Nat Methods* **9**, 255–258 (2012).

Mattes DR, Haynor H, Vesselle TK, Lewellyn W, Eubank. Nonrigid multimodality image registration in (International Society for Optics and Photonics, 2001), pp. 1609–1620.
